# Sex Differences in Behavioral and Brainstem Transcriptomic Neuroadaptations following Neonatal Opioid Exposure in Outbred Mice

**DOI:** 10.1523/ENEURO.0143-21.2021

**Published:** 2021-09-20

**Authors:** Kristyn N. Borrelli, Emily J. Yao, William W. Yen, Rhushikesh A. Phadke, Qiu T. Ruan, Melanie M. Chen, Julia C. Kelliher, Carly R. Langan, Julia L. Scotellaro, Richard K. Babbs, Jacob C. Beierle, Ryan W. Logan, William Evan Johnson, Elisha M. Wachman, Alberto Cruz-Martín, Camron D. Bryant

**Affiliations:** 1 Laboratory of Addiction Genetics, Departments of Pharmacology and Experimental Therapeutics and Psychiatry, Boston University School of Medicine, Boston, Massachusetts 02118; 2 Graduate Program for Neuroscience, Boston University, Boston, Massachusetts 02118; 3 Transformative Training Program in Addiction Science, Boston University, Boston, Massachusetts 02118; 4 NIGMS Training Program in Biomolecular Pharmacology, Boston University School of Medicine, Boston, Massachusetts 02118; 5 Neurobiology Section, Department of Biology, Boston University, Boston, Massachusetts 02215; 6 Molecular Biology, Cell Biology, and Biochemistry (MCBB), Boston University, Boston, Massachusetts 02215; 7 Undergraduate Research Opportunity Program, Boston University, Boston, Massachusetts 02118; 8 Laboratory of Sleep, Rhythms, and Addiction, Department of Pharmacology and Experimental Therapeutics, Boston University School of Medicine, Boston, Massachusetts 02118; 9 Center for Systems Neurogenetics of Addiction, The Jackson Laboratory, Bar Harbor, Maine 04609; 10 Department of Medicine, Computational Biomedicine, Boston University School of Medicine, Boston, Massachusetts 02118; 11 Department of Pediatrics, Boston University School of Medicine, Boston Medical Center, Boston, Massachusetts 02118

**Keywords:** neonatal abstinence syndrome, neonatal opioid withdrawal syndrome, opiate, opioid dependence, perinatal, rodents

## Abstract

The opioid epidemic led to an increase in the number of neonatal opioid withdrawal syndrome (NOWS) cases in infants born to opioid-dependent mothers. Hallmark features of NOWS include weight loss, severe irritability, respiratory problems, and sleep fragmentation. Mouse models provide an opportunity to identify brain mechanisms that contribute to NOWS. Neonatal outbred Swiss Webster Cartworth Farms White (CFW) mice were administered morphine (15 mg/kg, s.c.) twice daily from postnatal day 1 (P1) to P14, an approximation of the third trimester of human gestation. Female and male mice underwent behavioral testing on P7 and P14 to determine the impact of opioid exposure on anxiety and pain sensitivity. Ultrasonic vocalizations (USVs) and daily body weights were also recorded. Brainstems containing pons and medulla were collected during morphine withdrawal on P14 for RNA sequencing. Morphine induced weight loss from P2 to P14, which persisted during adolescence (P21) and adulthood (P50). USVs markedly increased at P7 in females, emerging earlier than males. On P7 and P14, both morphine-exposed female and male mice displayed hyperalgesia on the hot plate and tail-flick assays, with females showing greater hyperalgesia than males. Morphine-exposed mice exhibited increased anxiety-like behavior in the open-field arena on P21. Transcriptome analysis of the brainstem, an area implicated in opioid withdrawal and NOWS, identified pathways enriched for noradrenergic signaling in females and males. We also found sex-specific pathways related to mitochondrial function and neurodevelopment in females and circadian entrainment in males. Sex-specific transcriptomic neuroadaptations implicate unique neurobiological mechanisms underlying NOWS-like behaviors.

## Significance Statement

Neonatal opioid withdrawal syndrome (NOWS) is a poorly understood condition that has both a genetic and environmental component and is thought to be mechanistically distinct from opioid withdrawal in adults. The development of murine models for measuring neurobehavioral responses is critical for informing the neurobiological adaptations underlying NOWS. Using outbred mice that more closely model human genetic variation, we discovered several sex differences in behavioral timing and severity of NOWS model behaviors as well as transcriptomic adaptations in brain tissue that together suggest distinct mechanisms and sex-specific therapeutics for reversing withdrawal symptoms and restoring brain function.

## Introduction

The United States is in the midst of an opioid use disorder (OUD) epidemic. A recent report by the Centers for Disease Control and Prevention indicated a record 93,000 opioid-related deaths in 2020—a 29% increase from 2019 (https://www.cdc.gov). The OUD epidemic has been accompanied by a surge in infants exposed to opioids *in utero* (Jansson and Patrick, 2019) and a rise in rates of neonatal opioid withdrawal syndrome (NOWS; Milliren et al., 2018). NOWS is characterized by low birth weight, reduced weight gain, severe irritability, sleep fragmentation and restlessness, hypertonia, elevated pain sensitivity, and high-pitched cry, among other symptoms (Sutter et al., 2014). The current standard of treatment for NOWS includes tapered dosing of opioids over days or weeks, and other interventions that promote mother–infant bonding (Wachman et al., 2018; Ryan et al., 2019). Current treatments for NOWS primarily attenuate withdrawal severity for the infant and are believed to mitigate the potential long-term impact on mood, cognition, and pain. Further understanding of the impact of opioids on development is critical for the discovery, design, and implementation of new treatments and interventions for NOWS.

Several animal models for NOWS have been developed (Fodor et al., 2014; Chen et al., 2015; Byrnes and Vassoler, 2018), differing in time of exposure (i.e., prenatally, postnatally, or both), specific opioid (morphine, heroin, methadone, oxycodone, and/or buprenorphine), and dosing regimen (e.g., injections, pellets, mini-pumps; Byrnes and Vassoler, 2018; Robinson et al., 2020; Minakova et al., 2021). The physiological and behavioral consequences of opioids on development depends on the timing, duration, and route of drug administration (Fodor et al., 2014; Byrnes and Vassoler, 2018). In rodents, the period between postnatal day 1 (P1) through P14 is an approximation of the third trimester of human gestation (Barr et al., 2011), characterized by rapid, extensive synaptogenesis and myelination in the brain (Semple et al., 2013). For example, morphine administration between P1 and P14 (Robinson et al., 2020) in mice induced neurodevelopmental delays and effects on behaviors later during adolescence and adulthood, including marble burying and morphine locomotor sensitization behaviors (Robinson et al., 2020). Early neonatal opioid exposure in rodents has the following several key advantages: (1) opioid exposure during third trimester is associated with a higher risk for NOWS in human neonates (Desai et al., 2015), providing strong translational relevance; (2) neonatal exposure during the third trimester equivalent is sufficient to produce symptoms of withdrawal in mice and rats (Jones and Barr, 1995, 2001; McPhie and Barr, 2009; Robinson et al., 2020); (3) postnatal delivery of opioids enables accurate dosing to each pup; (4) avoiding maternal opioid exposure reduces the potential variance introduced by opioid-dependent alterations in maternal caretaking behaviors; and (5) the short-term and long-term effects of opioids on the brain and behavior can be assigned to a specific developmental period using discrete, timed administration of opioids.

Given these advantages, in the present study, we used an administration regimen similar to that previously described (Robinson et al., 2020) to further investigate the consequences of neonatal opioid exposure in mouse model of NOWS, including ultrasonic vocalizations (USVs), anxiety, and pain behaviors. To gain insight into the neurobiological adaptations related to NOWS, we also investigated the transcriptional alterations in the brainstem (medulla plus pons), a region of the brain implicated in neonatal opioid withdrawal (Jones and Barr, 2001; Maeda et al., 2002; McPhie and Barr, 2009) and hyperalgesia (Bederson et al., 1990; Kaplan and Fields, 1991; Vanderah et al., 2001; Ossipov et al., 2005; Xie et al., 2005; Vera-Portocarrero et al., 2011). We conducted our studies in outbred Cartworth Farms White (CFW) mice (Swiss Webster; both males and females) because of their greater genetic heterozygosity and increased number of historical recombination events compared with inbred strains and short-term (e.g., F2) intercrosses between them. These characteristics more closely model the complex genetic architecture of the human genome (Tuttle et al., 2018) and can be leveraged in future studies to conduct high-resolution genetic mapping of NOWS-related phenotypes (Gonzales and Palmer, 2014; Parker et al., 2016; Gonzales et al., 2018).

## Materials and Methods

### Mice

All experiments in mice were conducted in accordance with the National Institutes of Health *Guide for the Care and Use of Laboratory Animals* and were approved by the Institutional Animal Care and Use Committee. Outbred CFW mice (Swiss Webster) were purchased from Charles River Laboratory at 8 weeks of age. Breeders were paired after 1 week of habituation to the vivarium. Each breeder mouse was from a different litter, thus minimizing relatedness within a breeder pair (Parker et al., 2016). Laboratory chow (Teklad 18% Protein Diet, Envigo) and tap water were available *ad libitum.* Breeder cages were provided with nestlets. A maximum of three sequential litters per breeder were used in the study. Mice were maintained on a 12 h light/dark cycle (lights on, 6:30 A.M.). Phenotyping was conducted during the light phase between 9:00 A.M. and 12:00 P.M. A power analysis based on the effect size of the sex-averaged thermal nociceptive response on the hot plate on P7 in morphine-exposed mice versus saline (SAL) control mice (Cohen’s *d* = 1.55) was used to estimate the required sample size using a two-tailed *t* test in G*Power 3 (Faul et al., 2007). A sample size of 12 per treatment provides 95% power (*p* < 0.05). Thus, for the behavioral studies, we used a minimum of *n* = 12 per treatment (6 females, 6 males). All behavioral data were generated by female experimenters, thus controlling for the sex of the experimenter as a source of variance (Bohlen et al., 2014; Sorge et al., 2014).

### Morphine treatment regimen from P1 through P14

Morphine sulfate pentahydrate (Sigma-Aldrich) was dissolved in sterilized physiological saline (0.9%) in a 0.75 mg/ml morphine solution for systemic administration via injections (15 mg/kg, s.c., in 20 μl/g volume). The morphine dose is based on the total molecular weight of morphine sulfate pentahydrate (not just morphine). For P1 through P14, injections were administered twice daily mostly at 7:30 A.M. and 4:00 P.M. The only exceptions were at P7 and P14, at which time injections were administered at 10:00 A.M. and 4:00 P.M. to provide time for phenotypic assessment of spontaneous morphine withdrawal from 8:00 to 10:00 A.M. (see below). We used a mixed-litter design whereby one-half of the pups was randomly assigned to saline (20 μl/g, s.c.) and the other half was randomly assigned to morphine (15 mg/kg, s.c.) within the same litter. The rationale for choosing a mixed-litter design was to avoid introducing confounds between litters that could skew the results (e.g., genetic relatedness, maternal behavior, or the general cage environment). On P7, pups underwent behavioral phenotyping, as described below, 16 h following morphine administration. Systemically administered morphine reaches a peak plasma concentration at 15 min with a half-life of ∼37 min (Boasen et al., 2009). Thus, the behavioral effects associated with prior morphine exposure are attributed to withdrawal at 16 h following the last administration, rather than the acute, bioactive effects of morphine. Twice daily administration of morphine then continued until P14 when pups underwent behavioral phenotyping, followed by morphine administration at 10:00 A.M. and 4:00 P.M. On P15, 16 h after final morphine administration, mice were killed at 10:00 A.M. by live, rapid decapitation, and brain tissue was immediately harvested.

### General procedures

Experimenters (all females) were blinded to saline or morphine conditions during behavioral testing on P7 and P14. Pups were habituated to the testing room for at least 1 h before testing. Immediately before testing, pups were transferred to a new cage containing a heat pad. Dams and sires remained in their home cages in the testing room while the pups were tested. Activity in sound-attenuating chambers during ultrasonic vocalization recording and on the hot plate were video recorded using infrared cameras (Swann) and tracked with ANY-maze tracking software (Stoelting).

### Ultrasonic vocalizations in P7 and P14 mice

During maternal separation, withdrawal from repeated morphine administration increases USVs in rat pups (Barr and Wang, 1992). Before injections on P7 and P14, each mouse pup was placed into a Plexiglas box (43 cm length × 20 cm width × 45 cm height; Lafayette Instruments) placed within sound-attenuating chambers (Med Associates). USVs were recorded using Ultrasound Recording Interface (UltraSoundGate 816H, Avisoft Bioacoustics), and the concomitant distance traveled was calculated over 10 min. After 10 min, pups were removed from the boxes, weighed, and placed in a holding cage with a heating pad before nociceptive testing.

We used an unsupervised approach to categorize and compare syllable repertoires based on the similarity of their spectrotemporal patterns [mouse ultrasonic profile extraction (MUPET); Van Segbroeck et al., 2017]. Briefly, MUPET determines specific syllable types present in mouse USVs by analyzing their entire frequency structure, independent of syllable fundamental frequency or amplitude. Extracted syllable shapes were centered along time and frequency and were represented as low-dimension representations of USV patterns that captured their spectral shape and maintained the key spectrotemporal features (Bertrand et al., 2008; Joder and Schuller, 2012). Mean syllable duration was determined empirically from the distribution of syllable duration values in the saline group, then used for unsupervised analyses. Syllable thresholds were set at a minimum of 8 ms and a maximum of 200 ms. Optimal syllable repertoire size was determined using multiple measures from MUPET (Bayesian information criterion, average log likelihood, overall repertoire modeling score and goodness-of-fit; data not shown). The diagonal of the cross-repertoire similarity matrix, which provides the correlations between syllable unit pairs from two different repertoires, was used to compare the shapes of syllable units between experimental groups.

### Thermal nociception in P7 and P14 mice

Upon completion of USV recordings, the first pup was removed from the holding cage and placed in a Plexiglas cylinder (diameter, 15 cm; height, 33 cm) on a 52.5°C hot plate (IITC Life Science). On P7, the latency for the pup to turn onto their back was defined as the nociceptive response. The nociceptive response on P14 was the latency to flick or lick the forepaw or hindpaw, or jump. On P14, the majority of pups displayed a hindpaw lick. Pups were moved immediately following a pain response or following 30 s with no response. Immediately following nociceptive assessment on the hot plate, each pup was gently scruffed and the distal half of its tail was quickly lowered into a 48.5° hot water bath (LX Immersion Circulator, PolyScience) and the tail-flick latency was recorded.

### Tattooing in P7 mice

Following thermal nociception testing on P7, pups were tattooed for identification (ATS-3 General Rodent Tattoo System, AIMS). Pups were then injected with either morphine or saline and then returned to their home cage with the dam and sire.

### Self-righting in P4 and P7 mice

A subset of pups was also tested for self-righting as a developmental milestone (Robinson et al., 2020). On P4 and P7, each mouse was placed onto their back with all paws facing upward, and gently stabilized by a finger. The latency for the mouse to right itself and place all paws on the surface was recorded.

### Open field arena

A subset of mice underwent testing for anxiety-like behavior in the open field arena on P21. Mice could roam freely in Plexiglas boxes (20 cm width × 43 cm length × 46 cm height) for 5 min. Behavior was video recorded then later scored using ANY-maze software.

### Statistical analysis

Data analysis was performed in R (https://www.r-project.org/), SPSS Statistics 27 (IBM), and GraphPad Prism 8.0 (GraphPad Software). Body weight, hot plate, tail withdrawal, self-righting, USV/min, and USV distance data were analyzed using multifactorial repeated-measures (RM) ANOVAs, with drug treatment and sex as between-subject factors and either age/day or time as fixed-effects repeated measures. Distance and percentage of center time in the open field were analyzed using two-way ANOVA, with drug treatment and sex as independent variables. Body weight from P1 was also analyzed using a two-way ANOVA because of partially missing data. Before all ANOVAs, data residuals for each phenotype were assessed for normality before parametric analysis. In the case of a non-normal distribution of the residuals (failed Shapiro–Wilk test for normality, *p* < 0.05), data were percentile rank normalized as described previously (Templeton, 2011), and the Shapiro–Wilk test was repeated to confirm successful normalization (*p* > 0.05). To facilitate interpretation of the data, normalized data are presented as raw measurement values. Mauchly’s test of sphericity was performed for repeated measures with more than two levels (body weight and USV analysis). If the assumption of sphericity was violated, Greenhouse–Geisser-corrected statistics were reported. Homogeneity of variances was confirmed using Levene’s test of equality of error variances. *Post hoc* comparisons were performed in the case of a significant interaction to determine group differences. Either *t* tests (paired or unpaired) with Bonferroni correction or Tukey’s HSD tests were conducted for multiple comparisons.

### Brainstem RNA sequencing

On P15, 16 h following the final morphine injection (during a state of spontaneous morphine withdrawal), mice were killed, and brains were quickly removed to collect the brainstem containing the medulla and pons. Brainstems were transferred to tubes containing RNAlater were stored at 4°C, then 5 d later were lightly blotted and transferred to new tubes to be stored at −80°C until RNA extraction. RNA was extracted using TRIzol (Qiagen), ethanol precipitation, filtering columns (Qiagen), and elution with sterile, double-deionized water (Yazdani et al., 2015). Samples were diluted to 50 ng/μl. RNA library preparation (poly-A selection) and RNA sequencing (RNA-seq) was conducted at the University of Chicago Genomics Facility on an Illumina NovaSEQ 6000 sequencing system using a NovaSEQ SP-100 bp flowcell/reagent cassette. The 12 multiplexed, pooled samples (6 mice from saline and 6 mice from morphine groups) were sequenced (100 bp single-end) on a single lane of the two-lane flowcell. We used the R/Bioconductor package “scruff” for data preprocessing, including demultiplexing, read alignment, read counting, quality checking, and data visualization (Wang et al., 2019). Reads were trimmed for quality using Trimmomatic (Bolger et al., 2014). Trimmed reads were then aligned to the mm10 mouse reference genome (Ensembl) to generate BAM files for alignment using STAR (Dobin et al., 2013). The featureCounts read summarization program was used to count reads mapping to the “exon” feature in a GTF file obtained from Ensembl (GRCm38). Differential gene expression analysis of normalized read counts from Rsubread (Liao et al., 2019) was performed in R using the exactTest function in edgeR (Robinson et al., 2010). RIN (RNA integrity number) scores from the Bioanalyzer ranged from 8.2 to 8.9, indicating high-quality RNA for sequencing. We obtained an average of 43.7 million reads per sample (range, 35–60 million reads/sample); 97.7–98.6% of these reads had a perfect bar code match. Phred quality scores were all 36.4. Greater than 90% of the reads mapped uniquely to a single locus. FASTQ files and normalized read counts are available for download from the National Center for Biotechnology Information Gene Expression Omnibus (catalog #GSE141066).

### Gene network and pathway enrichment

Network analysis and plot generation were performed using Cytoscape software. Gene expression datasets [containing Log2 fold change (Log_2_FC) and *p* values] were uploaded for pathway analysis, and networks were plotted using known gene interactions imported from the STRING database. All genes with unadjusted *p* values < 0.10 were uploaded for network analysis, which enabled the detection of more broad patterns of gene expression and pathways within specific functional networks. We first analyzed the effect of morphine on gene expression within the combined analysis and included sex as a covariate. For the combined analysis, we plotted the top six GO (Gene Ontology) Biological Process networks that contained <50 genes to simplify viewing of the network plot and avoid overly broad enrichment terms. We then probed sex-specific effects of morphine by separately analyzing females and males, and determined the top KEGG enrichment terms and identified the gene–gene interactions as determined by STRING.

### Alternative splicing

Analysis was performed using the R package ASpli (Mancini et al., 2021; Bioconductor version 1.14.0). We first performed feature extraction of the GTF annotation files to retrieve information regarding gene structure, including intron and exon bins, junctions, and intron flanking regions. Using the BAM files, we mapped RNA-seq reads to the annotated genome to determine read count density for each extracted feature. We determined differential intron/exon bin usage to identify alternative splicing events. We assessed the main effect of morphine treatment on splicing events in sex-collapsed, female-only, and male-only datasets. We also identified splicing events influenced by interactive effects of condition and sex.

### Correlation of read counts with P14 behavioral phenotypes

To gain further insight into the molecular adaptations contributing to the behavioral adaptations induced by morphine, we examined the correlation between gene expression levels and phenotypes collected on P14. We specifically sought to identify genes that were differentially expressed in the morphine condition collapsed across sex and genes with normalized read counts that significantly correlated with P14 phenotypes (hot plate latency, tail withdrawal latency, USVs per minute, distance traveled during USV recordings, and body weights. Pearson product moment correlation coefficients were calculated between the normalized read counts and each of these phenotypes.

## Results

### Reduced weight gain following neonatal morphine exposure

**Figure 1.**
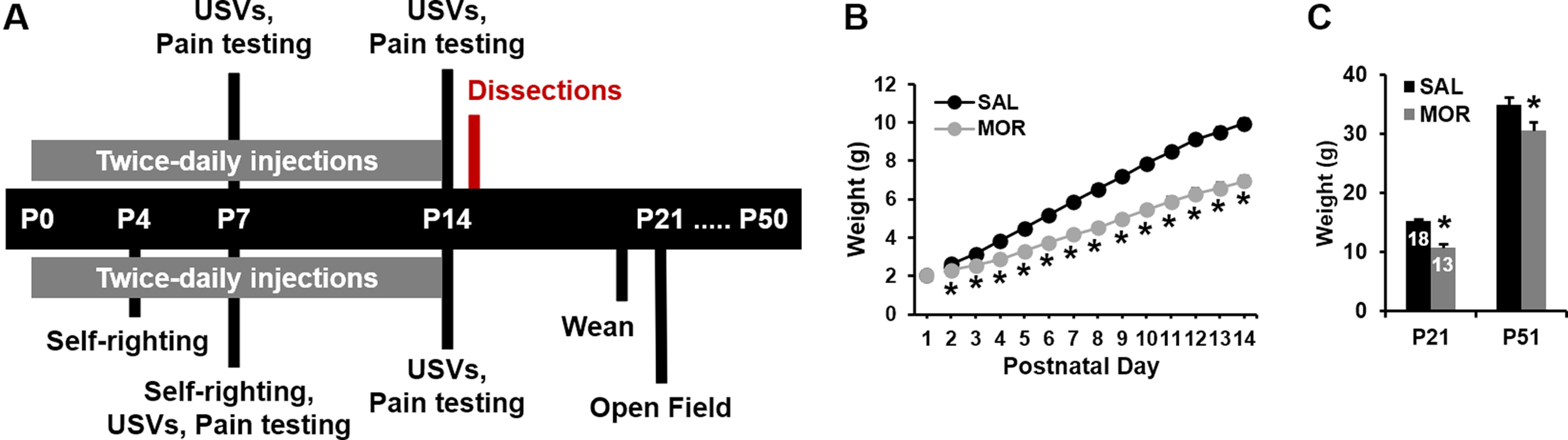
Experimental timeline for repeated neonatal opioid exposure and reduced weight gain. ***A***, Mice underwent assessment for USVs and thermal nociception. A subset of mice also underwent self-righting assessment at P4 and P7 and testing for locomotor activity and center time in the open field on P21. ***B***, In examining changes in body weight, a two-way ANOVA (treatment, sex) revealed no main effect of treatment on P1 (saline, *n* = 29; morphine, *n* = 25; *F*_(1,50)_ = 0.61, *p* = 0.44). Thus, body weight was not significantly different between treatment groups before the first injections. There was the expected main effect of sex (*F*_(1,50)_ = 11.10, *p* = 0.002) but no treatment × sex interaction (*F*_(1,50)_ = 0.78; *p* = 0.31). Body weight was subsequently analyzed from P2 to P14 via RM ANOVA, with treatment and sex as factors and postnatal day as the RM. Mauchly’s test indicated the assumption of sphericity had been violated (χ^2^_(77)_ = 1711.02, *p* < 0.001), so the Greenhouse–Geisser correction was applied (ε = 0.11). There was a main effect of treatment (saline, *n* = 41; morphine, *n* = 35; *F*_(1,72)_ = 98.04, *p* <0.001), postnatal day (*F*_(1.36,97.97)_ = 1526.99, *p* < 0.001), and treatment × postnatal day interaction (*F*_(1.36,97.97)_ = 72.61, *p* < 0.001), but no treatment × sex × postnatal day interaction (*F*_(1.36,97.97)_ = 0.07; *p* = 0.86); thus, the sex-combined data are presented. *Post hoc* pairwise *t* tests revealed significantly lower body weight in the morphine group (*Bonferroni-adjusted *t* tests; all *p* < 3 × 10^−4^; α_adjusted_ = 0.004). ***C***, In examining body weight in the subset of mice that was assessed at P21 and P51, RM ANOVA indicated a main effect of treatment (*F*_(1,27)_ = 29.09, *p* < 0.001), sex (*F*_(1,27)_ = 25.119, *p* < 0.001), postnatal day (*F*_(1,27)_ = 1174.26, *p* < 0.001), and postnatal day × sex interaction (*F*_(1,27)_ = 31.17, *p* < 0.001). In support of the treatment effect, morphine mice had significantly lower body weight compared with saline mice at both P21 (*t_(1,29)_ = −7.33; *p* < 0.001) and P50 (*t_(1,29)_ = −2.27; *p* = 0.03).

A schematic of the timeline for phenotyping is provided in Figure 1*A*. Hallmarks of NOWS in human neonates are low birth weight and reduced weight gain. On P1, before morphine administration, body weights were similar across mice (Fig. 1*B*; saline, *n* = 29; morphine, *n* = 25). By P2, pups that were administered morphine (*n* = 35) already had significantly lower normalized body weights relative to saline pups (*n* = 41), and this effect persisted through P14 (Fig. 1*B*). Lower-body weights were also evident in morphine-exposed pups (*n* = 18) compared with the saline group (*n* = 18) at P21 and P51 in a subset of mice tested for self-righting and open field behavior (Fig. 1*C*). Body weights for pups that died before P14 (6 of 41 morphine-exposed pups, ∼15%) were excluded from further analyses. Pups were identified as female or male on P14, so sex-specific mortality could not be determined. No deaths were observed in the saline group. Thus, morphine administration from P1 to P14 led to acute reductions in body weights and marked lower weight gain that persisted into adulthood.

### Altered self-righting latency in a sex-specific and development-dependent manner following repeated neonatal morphine exposure

**Figure 2.**
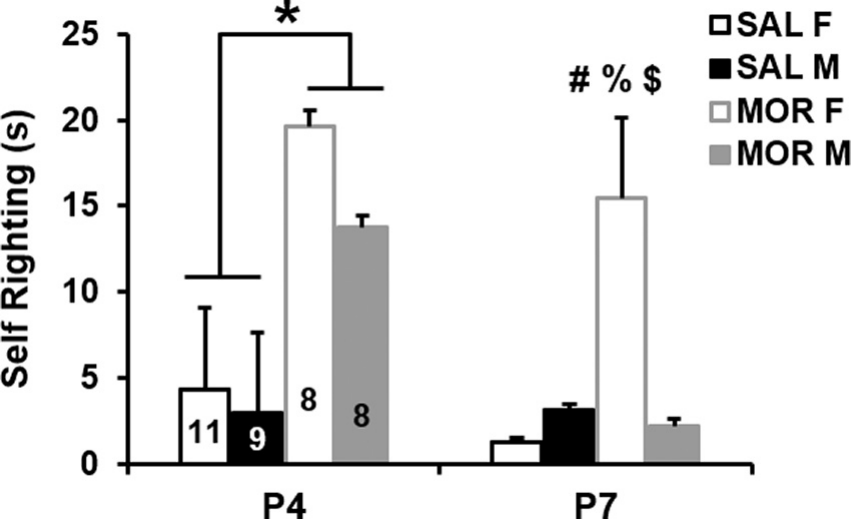
Altered self-righting latency in a sex-specific and development-dependent manner following repeated neonatal morphine exposure. In examining the normalized latency to self-right on P4 and P7, RM ANOVA indicated a main effect of treatment (*F*_(1,32)_ = 49.23; *p* < 0.001), postnatal day (*F*_(1,32)_ = 18.61, *p* < 0.001), and sex (*F*_(1,32)_ = 8.30, *p* = 0.007), as well as treatment × sex (*F*_(1,32)_ = 4.29, *p* = 0.046) and treatment × sex × postnatal day interactions (*F*_(1,32)_ = 4.69, *p* = 0.038). Data from each day were then analyzed separately. On P4, two-way ANOVA indicated a main effect of treatment (*F*_(1,32)_ = 14.09; *p* < 0.001), as morphine-treated mice took 8.47 s longer to self-right on average (95% CI for difference: [4.03, 12.91]; *p < 0.001). On P7, two-way ANOVA indicated a main effect of treatment (*F*_(1,32)_ = 24.24, *p* < 0.001) and a treatment × sex interaction (*F*_(1,32)_ = 8.19; *p* = 0.007) that was explained by significantly longer latencies to self-right in morphine females compared with all other groups: versus saline females (95% CI for difference: [9.64, 27.52]; #*p* < 0.001); versus saline males ([6.66, 25.36]; %*p* < 0.001), and versus morphine males ([1.47, 20.71]; $*p* = 0.02). Morphine-exposed males did not differ significantly from saline control males (*p* = 0.49) or females (*p* = 0.12). Data are presented as the mean ± SEM of raw (non-normalized) data.

Self-righting from back to abdomen is used to test milestones associated with motor development. On P4, pups administered morphine displayed significantly prolonged normalized latencies to self-right compared with saline mice (Fig. 2). By P7, females administered morphine still exhibited prolonged self-righting latencies compared with all other groups, including morphine-treated males (Fig. 2). Together, our results suggest a morphine-induced developmental delay that was more persistent in morphine-exposed females compared with morphine-exposed males.

### Increases in USVs during maternal separation following repeated neonatal morphine exposure

**Figure 3.**
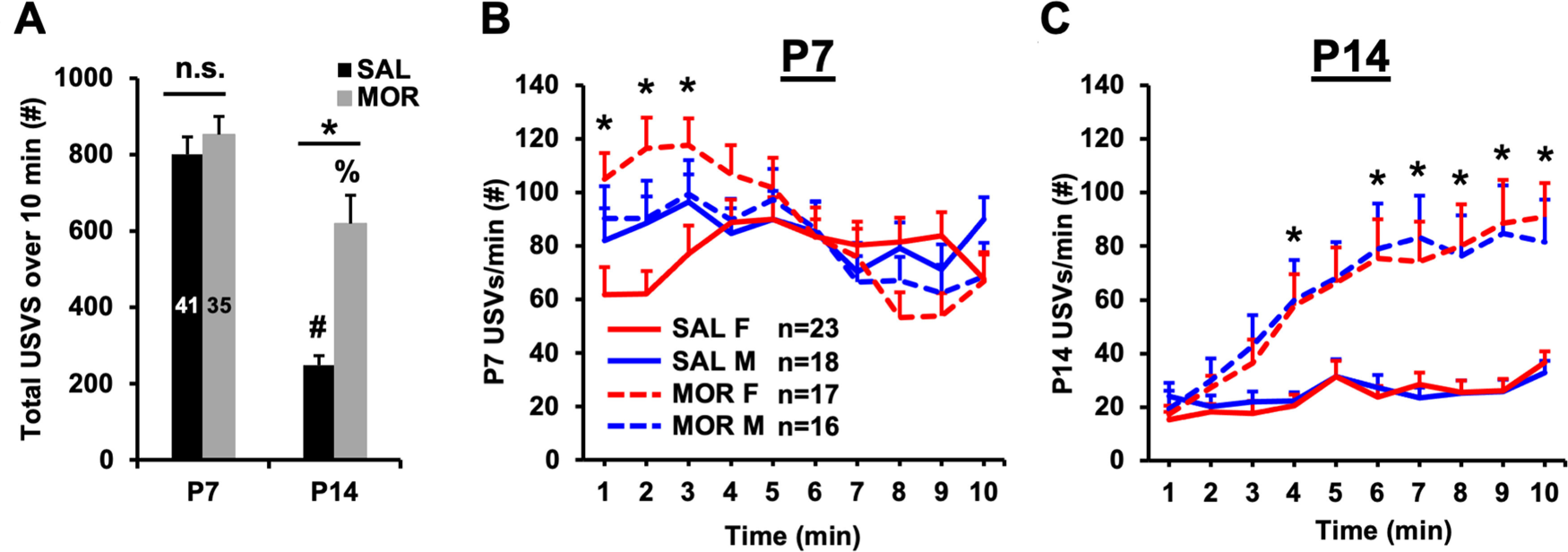
Pronounced increases in USVs during maternal separation following repeated neonatal morphine exposure. ***A***, In examining normalized total USVs emitted over 10 min, RM ANOVA (treatment and sex as factors, postnatal day as the RM) revealed a main effect of treatment (*F*_(1,69)_ = 8.77, *p* = 0.004), postnatal day (*F*_(1,69)_ = 70.89, *p* < 0.001), and treatment × postnatal day interaction (*F*_(1,69)_ = 5.10, *p* = 0.03). There was no effect of sex (*F*_(1,69)_ = 0.05, *p* = 0.82) and no interactions with sex (all *p* > 0.37). Tukey’s *post hoc* comparisons revealed no difference between treatment groups on P7 [95% CI for difference (saline – morphine): [−236.51, 118.56]; *p* = 0.82]. On P14, however, morphine-treated mice emitted more USVs than saline-treated mice [95% CI for difference (saline – morphine): [−531.81, −173.80]; *p < 0.001]. Both the saline- and morphine-treated groups showed a significant reduction in USVs emitted from P7 to P14 (saline group mean difference = −555.40, #*p* < 0.001; morphine group mean difference = −261.56, %*p* = 0.002). ***B***, Greenhouse–Geisser corrections were applied when examining normalized USVs per minute on both P7 and P14 (Mauchly’s test; P7: χ^2^_(44)_ = 117.39, *p* < 0.001, ε = 0.69; P14: χ^2^_(44)_ = 155.41, *p* < 0.001, ε = 0.60). On P7, RM ANOVA (treatment and sex as factors, time as the RM) identified a treatment × sex × time interaction (*F*_(6.22,435.22)_ = 2.34; *p* < 0.03). Subsequent RM ANOVA of females only revealed a significant treatment × time interaction (*F*_(6.07,230.58)_ = 6.91; *p* < 0.001) that was driven by elevated USVs per minute during the first 3 min in morphine-exposed females compared with saline-control females (*Bonferroni adjusted: *p* = 0.004, 0.009, and 0.02, respectively). In contrast, RM ANOVA of males only indicated no significant treatment × time interaction (*F*_(5.29,169.19)_ = 0.83; *p* = 0.54) but only a main effect of time (*F*_(5.29,169.19)_ = 3.54; *p* = 0.004). ***C***, In examining normalized USVs per minute on P14, RM ANOVA (treatment and sex as factors, time as the RM) indicated main effects of treatment (*F*_(1,70)_ = 14.09, *p* < 0.001) and time (*F*_(5.41,378.88)_ = 34.01, *p* < 0.001), and a treatment × time interaction (*F*_(5.41,378.88)_ = 8.19, *p* < 0.001). There was no main effect or interaction with sex (all *p* values > 0.47). We performed unpaired *t* tests between treatment groups (collapsed across sexes) to determine group differences across the 10 min testing period. For minutes 4 and 6–10, morphine-exposed pups emitted significantly more USVs per minute than saline-control pups (*all Bonferroni-adjusted *p* values < 0.03). At minute 5, the same trend was present, but was not significant (adjusted *p* = 0.07). Data are presented as the mean ± SEM of raw (non-normalized) data.

In rodents, USVs are thought to model a negative affective state and were used as an indicator of opioid withdrawal (Barr et al., 2011). Before running ANOVAs, USV data (cumulative USVs, P4 USVs per minute, and P7 USVs per minute) were percentile rank normalized (Templeton, 2011). We found complex relationships among treatment, sex, age, and time in the USV data. As expected, control pups receiving saline displayed an overall developmental decrease in USVs from P7 through P14 (Noirot, 1966; Fig. 3*A*). Notably, morphine-exposed pups emitted more USVs compared with saline pups on P14, but not on P7 (Fig. 3*A*), which could either be explained by a developmental delay or by spontaneous morphine withdrawal state.

We then analyzed the effects of morphine treatment and sex on USVs in a time-dependent manner (USVs per minute over a 10 min recording period). On P7, morphine-exposed females emitted significantly more USVs per minute than saline control females during the first 3 min of the recording session (Fig. 3*B*). In the males-only analysis, however, there was no significant effect of morphine treatment. On P14, we detected significant elevations in USVs per minute in morphine-treated pups during minute 4 and minutes 6–10 of the recording session (Fig. 3*C*). Unlike on P7, on P14, we did not detect significant effects of sex or interactions of sex with other factors. Our findings indicate that neonatal morphine exposure from P1 to P14 results in elevated USV rates in females that emerge early in the testing session on P7, as well as sex-independent increases in USV rates that emerge during the latter half of the 10 min testing period on P14.

### Concomitant locomotor activity during USV assessment

**Figure 4.**
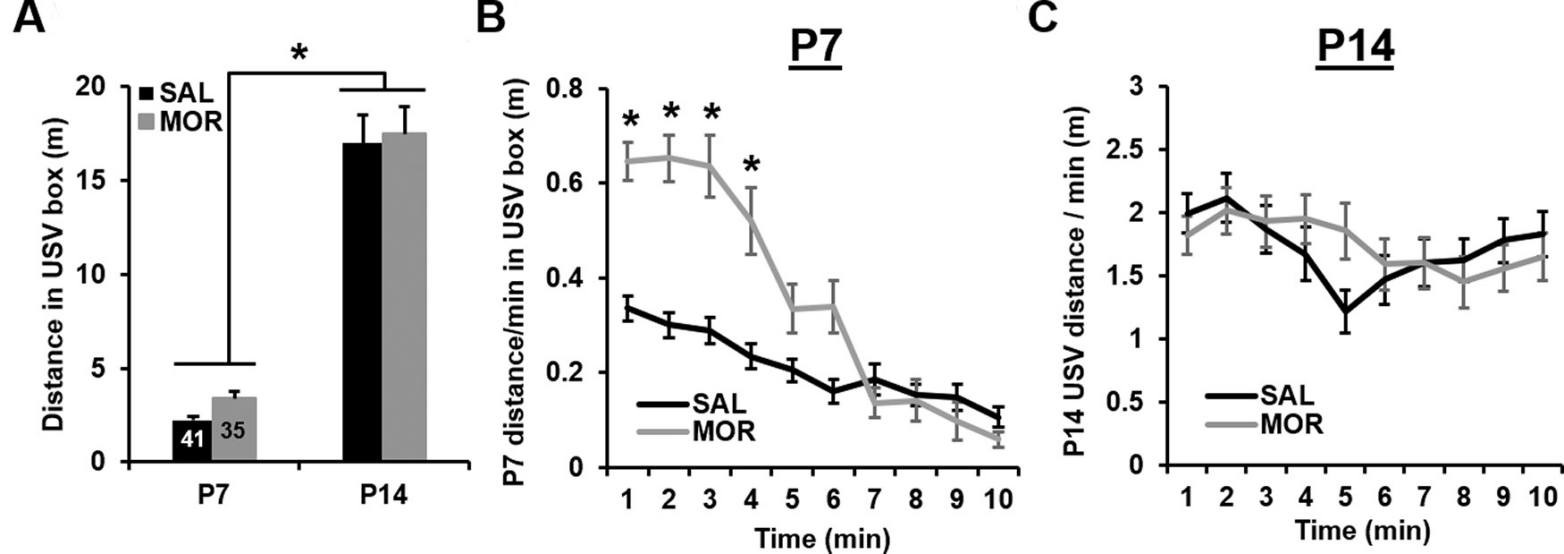
Concomitant locomotor activity during USV recordings following repeated neonatal morphine exposure. ***A***, RM ANOVA of normalized total distance during USV recordings (treatment and sex as factors, postnatal day as the RM) indicated no effect of treatment (*F*_(1,70)_ = 0.78; *p* = 0.38), but there was a significant effect of postnatal day (*F*_(1,70)_ = 199.52, *p < 0.001), as pups traveled an average of 14.36 ± 1.02 m further on P14 versus P7. There was no effect of sex (*F*_(1,70)_ = 0.26, *p* = 0.61), and there were no significant interactions (all *p* values ≥ 0.59). ***B***, To detect any time-dependent effects of morphine on locomotor activity during USV recordings, we also assessed distance traveled per min. Again, data were normalized before parametric analysis and Greenhouse–Geisser corrections were applied (Mauchly’s test; P7: χ^2^_(44)_ = 135.38, *p* < 0.001, ε = 0.67; P14: χ^2^_(44)_ = 233.39, *p* < 0.001, ε = 0.52). On P7, RM ANOVA (treatment and sex as factors, time as the RM) indicated the main effects of treatment (*F*_(1,71)_ = 0.67, *p* = 0.04) and time (*F*_(6.03,428.15)_ = 77.96, *p* < 0.001), and significant treatment × time (*F*_(6.03,428.15)_ = 15.81, *p* < 0.001) and treatment × sex (*F*_(1,71)_ = 4.29, *p* = 0.04) interactions. The time × treatment interaction was driven by increased distance traveled by morphine-exposed pups during minute 1 through minute 4 of USV recordings (*all adjusted *p* values ≤ 0.02; unpaired *t* tests with Bonferroni correction). The treatment × sex interaction was driven by an increase in distance traveled in morphine-exposed males relative to saline males (0.12 ± 0.04 m; Bonferroni-adjusted *p* = 0.006). ***C***, On P14, RM ANOVA (treatment and sex as factors, time as the RM) indicated a main effect of time (*F*_(4.7,333.70)_ = 4.86, *p* < 0.001) and a near-significant time × treatment interaction (*F*_(4.7,333.70)_ = 2.21, *p* = 0.057). There was no main effect of sex or interactions with sex (*p* values > 0.23). In contrast to P7, there was no significant difference between saline control and morphine-exposed mice at any time point (all Bonferroni-adjusted *p* values ≥ 0.53). Data are presented as the mean ± SEM of raw (non-normalized) data.

While there was no effect of prior morphine exposure on normalized total distance, pups in both treatment groups traveled significantly farther on P14 relative to P7, which is likely attributable to normal motor development and increased mobility between these developmental timepoints (Fig. 4*A*). Analyzing average velocity (normalized distance per minute) over the 10 min recording period on P7 revealed significant time-dependent differences between treatment groups, with morphine pups displaying higher velocities during the first 4 min of USV recordings relative to saline controls (Fig. 4*B*). Additionally, independent of time, morphine males had higher average velocities than saline males on P7. Unlike on P7, on P14, there were no significant differences between treatment groups on P14 (treatment × time, *p* = 0.057; Fig. 4*C*).

### Altered USV syllable repertoires following repeated neonatal opioid exposure

**Figure 5.**
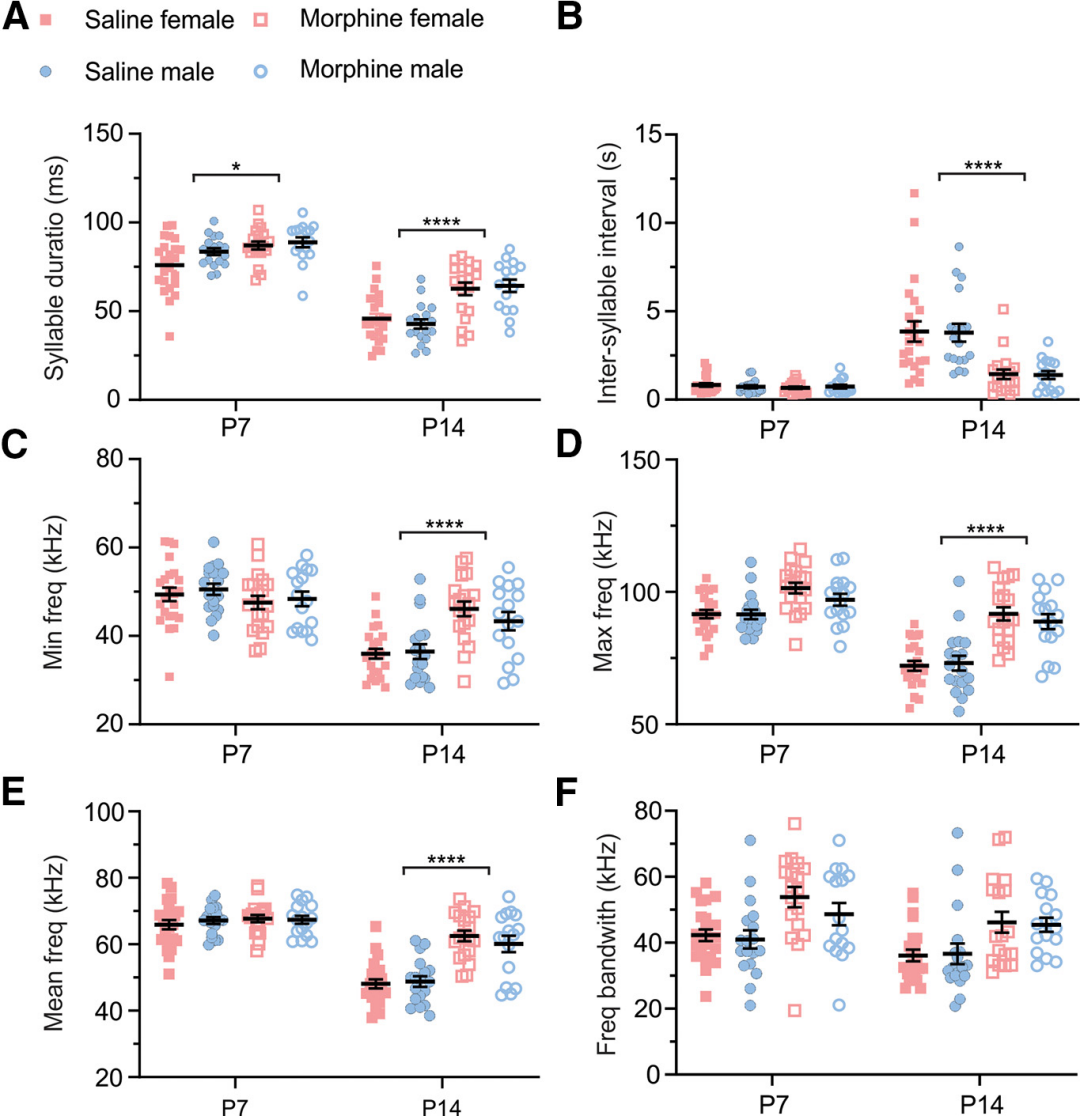
Changes in ultrasonic vocalizations following repeated neonatal morphine exposure. ***A***, There was an increase in normalized syllable duration in morphine-exposed females and males on P14 relative to saline controls. There were main effects of treatment (*F*_(1,40)_ = 42.97, ****p <* *0.0001), postnatal day (*F*_(1,40)_ = 240.0, ****p <* *0.0001), and a significant treatment × postnatal day interaction (*F*_(1,28)_ = 6.15, *p =* *0.02). There was no main effect of sex (*F*_(1,72)_ = 0.75, *p *=* *0.39) or any interactions with sex (treatment × sex: *F*_(1,72)_ = 0.004, *p *=* *0.95; age × treatment × sex: *F*_(1,72)_ = 1.09, *p *=* *0.30). Tukey’s *post hoc* comparisons revealed the following: P7: saline versus morphine, *p =* *0.0249; P14: saline versus morphine, ****p <* *0.0001. ***B***, Repeated morphine exposure led to a decrease in normalized intersyllable interval in female and male P14 mice relative to saline controls. There were main effects of treatment (*F*_(1,40)_ = 60.06, ****p <* *0.0001) and postnatal day (*F*_(1,40)_ = 138.3, ****p <* *0.0001) as well as a significant treatment × postnatal day interaction (*F*_(1,28)_ = 54.82, ****p <* *0.0001). Again, there were no main effects of sex (*F*_(1,144)_ = 0.15, *p* = 0.69) or any interactions with sex (treatment × sex: *F*_(1,144)_ < 0.001, *p *=* *0.9854; treatment × postnatal day × sex: *F*_(1,144)_ = 0.08, *p* = 0.77). Tukey’s *post hoc* comparisons revealed the following: P7: saline versus morphine, *p *=* *0.99; P14: saline versus morphine, ****p < 0.0001. ***C***, Chronic opioid exposure led to an increase in normalized minimum frequency in female P14 mice relative to saline controls. There was a main effect of postnatal day (*F*_(1,40)_ = 192.10, ****p <* *0.0001) and a significant treatment × postnatal day interaction (*F*_(1,28)_ = 25.55, ****p <* *0.0001). There was no main effect of treatment (*F*_(1,40)_ = 3.489, *p* = 0.0691), no main effect of sex (*F*_(1,72)_ < 0.001, *p* = 0.98), and no interactions with sex (treatment × sex: *F*_(1,72)_ = 0.22, *p *=* *0.64; treatment × postnatal day × sex: *F*_(1,72)_ = 0.46, *p* = 0.50). Tukey’s *post hoc* comparisons revealed the following: P7: saline versus morphine, *p *=* *0.28; P14: saline versus morphine, ****p <* *0.0001. ***D***, Repeated morphine exposure led to an overall increase in normalized maximum frequency relative to saline controls. There were main effects of treatment (*F*_(1,40)_ = 71.76, ****p <* *0.0001) and postnatal day (*F*_(1,40)_ = 71.08, ****p <* *0.0001) and a significant treatment × postnatal day interaction (*F*_(1,28)_ = 29.02, ****p <* *0.0001). There was no main effect of sex (*F*_(1,72)_ = 0.96, *p* = 0.33), and there were no interactions with sex (treatment × sex: *F*_(1,72)_ = 1.11, *p* = 0.30; treatment × postnatal day × sex: *F*_(1,72)_ = 0.10, *p* = 0.76). Tukey’s *post hoc* comparisons revealed the following: P7: saline versus morphine, *p *=* *0.13; P14: saline versus morphine, ****p <* *0.0001. ***E***, Chronic opioid exposure led to an increase in normalized mean frequency in female and male P14 mice relative to saline controls. There were main effects of treatment (*F*_(1,40)_ = 42.90, ****p <* *0.0001) and postnatal day (*F*_(1,40)_ = 107.1, ****p <* *0.0001), and a significant treatment × postnatal day interaction (*F*_(1,28)_ = 33.73, ****p <* *0.0001). There was no main effect of sex (*F*_(1,72)_ = 0.06, *p* = 0.82), and there were no interactions with sex (treatment × sex: *F*_(1,72)_ = 0.78, *p* = 0.38; treatment × postnatal day × sex: *F*_(1,72)_ = 0.16, *p* = 0.69). Tukey’s *post hoc* comparisons revealed the following: P7: saline versus morphine, *p *=* *0.95; P14: saline versus morphine, ****p <* *0.0001. ***F***, Chronic opioid exposure led to an overall increase in frequency bandwidth across groups relative to saline controls. There were main effects of both treatment (*F*_(1,40)_ = 28.50, ****p <* *0.0001) and postnatal day (*F*_(1,40)_ = 13.98, ***p =* *0.0006), with no significant treatment × postnatal day interaction (*F*_(1,28)_ = 1.676, *p* = 0.2068). There was no main effect of sex (*F*_(1,72)_ = 0.4988, *p* = 0.4823), and there were no interactions with sex (treatment × sex: *F*_(1,72)_ = 0.1259, *p* = 0.7238; treatment × postnatal day × sex: *F*_(1,72)_ = 0.3515, *p* = 0.5551). For all parameters, we performed a three-way ANOVA mixed-effects model followed by a Tukey’s *post hoc* test.

We used MUPET (Van Segbroeck et al., 2017) to determine whether morphine exposure during early postnatal development caused changes in the spectrotemporal patterns of USV syllables. Our analysis showed an overall difference in the USV spectrotemporal patterns between P7 and P14 groups (Fig. 5; age, ***p < 0.001 for all parameters tested), suggesting that the USVs were developmentally regulated. In support of this observation, in saline controls, there was a developmental decrease in syllable duration (Fig. 5*A*; ∼43.43%) and an increase in intersyllable interval (Fig. 5*B*; ∼4.9-fold change). Additionally, in P14 saline controls, there was an overall decrease in minimum frequency (Fig. 5*C*; ∼27.47%), maximum frequency (Fig. 5*D*; ∼20.82%), mean frequency (Fig. 5*E*; ∼27.14%), and frequency bandwidth (Fig. 5*F*; ∼12.90%). Overall, we did not observe effects of sex or interactions with sex in our analysis (Fig. 5*A–F*), suggesting that the USV spectrotemporal patterns were not different between females and males.

For all the parameters measured (Fig. 5*A–F*), we observed an effect of treatment, suggesting that repeated morphine exposure altered the spectrotemporal patterns of USVs, relative to saline controls. Additionally, we observed an effect of postnatal day × treatment in all but one parameter measured [Fig. 5*A–E* (but not Fig. 5*F*)], suggesting that alterations in spectrotemporal USV patterns as a results of prior morphine exposure depend on postnatal day. In support, *post hoc* analysis indicated that for several parameters, repeated morphine administration had the most robust effects on P14 (Fig. 5*A–F*; syllable duration, intersyllable interval, maximum frequency, and mean frequency; saline vs chronic opioid exposure, ***p <* *0.001), relative to P7 timepoints. Last, for all the parameters measured (Fig. 5*A–F*), we did not observe any treatment × sex interactions or any treatment × sex × day interactions. Together, these data suggest that USV spectrotemporal patterns are developmentally regulated and are altered by repeated morphine exposure, regardless of sex.

**Figure 6.**
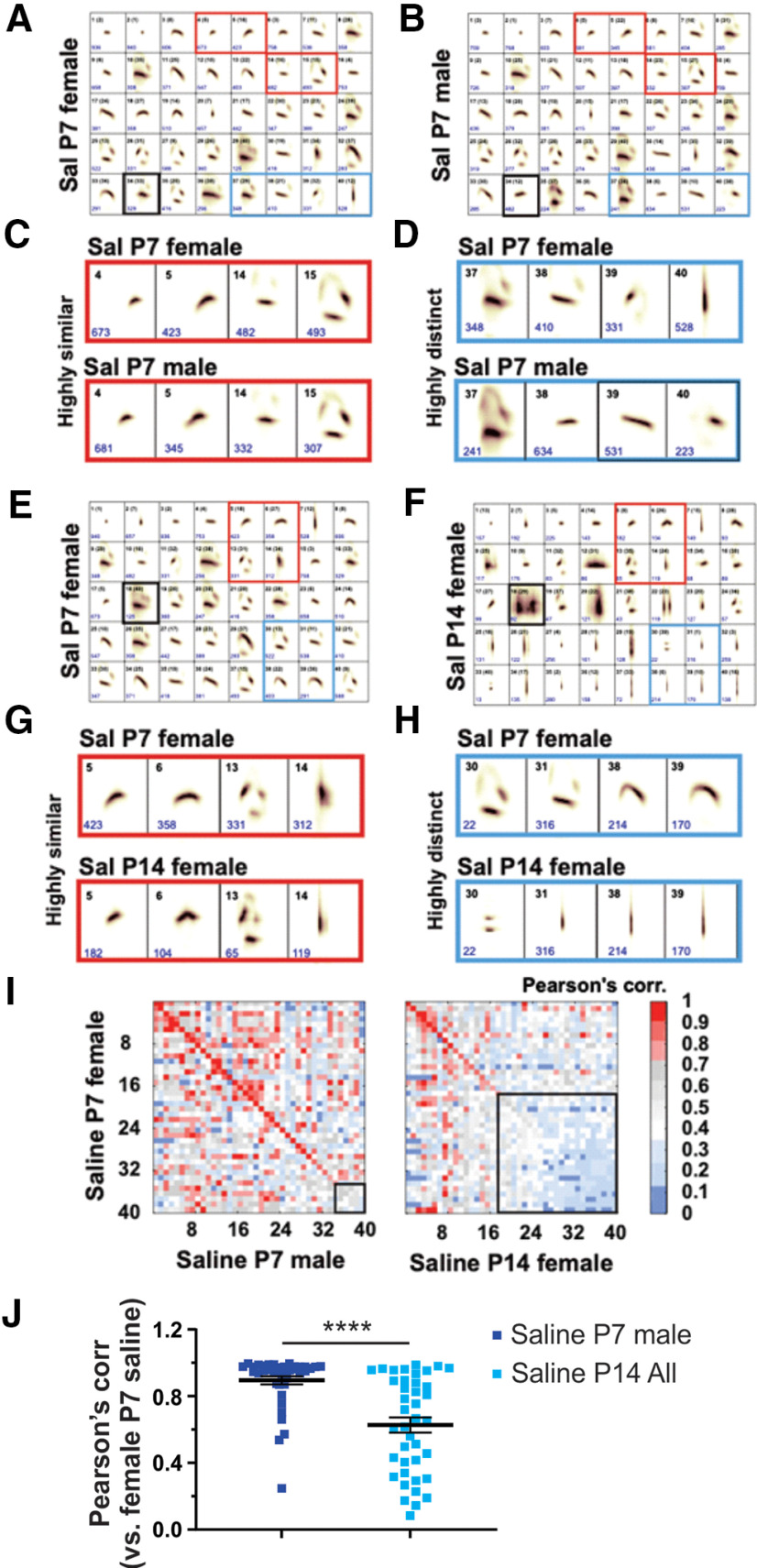
USV spectrotemporal structure is developmentally regulated from the first postnatal week (P7) to the second postnatal week (P14). ***A***, ***B***, Repertoires showing the 40 syllable types (average shape; top black numbers, syllable number) learned from processing recordings across the P7 saline datasets. Syllable types are listed in order of frequency of use from left to right (1–40), with the total number of syllables that are present in each syllable unit given in blue. Black number in parenthesis, original syllable number from datasets before performing comparison analysis. Red and blue squares are examples of highly similar or distinct syllables across groups, respectively. Pearson similarity scores are <0.70 after syllable labeled with bold black square. ***C***, ***D***, Examples of highly similar (***A***, ***B***, red squares) and distinct syllables across saline P7 groups. ***E***, ***F***, Same as in ***A*** and ***B***, but syllables learned from saline P7 and P14 datasets. ***D–G***, Examples of highly similar (***A***, ***B***, red squares) and distinct syllables across saline P7 groups. ***A–H***, The lower side of each syllable frame is 150 ms. ***I***, Similarity matrix of the spectral types of pairs of syllable units learned from comparisons between reference groups (saline P7 females) and saline P7 male mice (left panel) or saline P14 female mice (right panel). The matrix diagonal gives the Pearson correlation for sequential pairs of syllables from compared datasets ranked from most to least similar (e.g., syllable 1 in both repertories are highly similar). Bold black square in the similarity matrix indicates highly distinct syllable units (Pearson score, ≤0.70) and increases in saline P7/P14 comparison (left panel) relative to P7 comparisons (right panel), suggesting developmental regulation of USV spectrotemporal structure. ***I***, ***J***, Mean Pearson correlation values obtained from the diagonal in comparisons of saline P7 male (***I***, left) and P14 groups of both sexes (***I***, right; Fig. 7*C*) to saline P7 female mice, the “Reference Group.” Red and blue values, Pearson correlations for highly similar and distinct pairs of syllables, respectively. Saline P7 males versus saline P14 sex-combined (“All”). ****p <* *0.0001, *t* test. Data are presented as the mean ± SEM.

We next categorized syllable repertoires in female and male mice administered saline (controls) and arranged repertoires based on spectrotemporal similarity (Fig. 6*A****–****H*). We found that although syllable units between female and male saline mice were overall highly similar on P7 (Pearson’s correlation, 0.90 ± 0.02; Fig. 6*I*, left, *J*), there were some subsets of syllable repertoires that were highly similar or distinct (Fig. 6*A–D*, red and blue squares, respectively). In contrast to the relative similarity found between the sexes at P7, Pearson’s correlation values indicated a more robust difference in syllable repertoires between the reference group (saline females at P7) and both the saline female group and the saline male group at P14 (Figs. 6*E–I*, right, *J*, 7*C*; values obtained from the diagonal; P7 saline female group vs P14 saline female and male groups, Pearson’s correlation, ≤0.70). Overall, our findings suggest that independent from morphine exposure, our approach had the sensitivity to detect developmental changes in the spectrotemporal patterns of USV syllable repertoires of early postnatal mice. In addition, these results suggest a transition in the spectrotemporal structure of USV syllables during the first 2 weeks of postnatal development.

**Figure 7.**
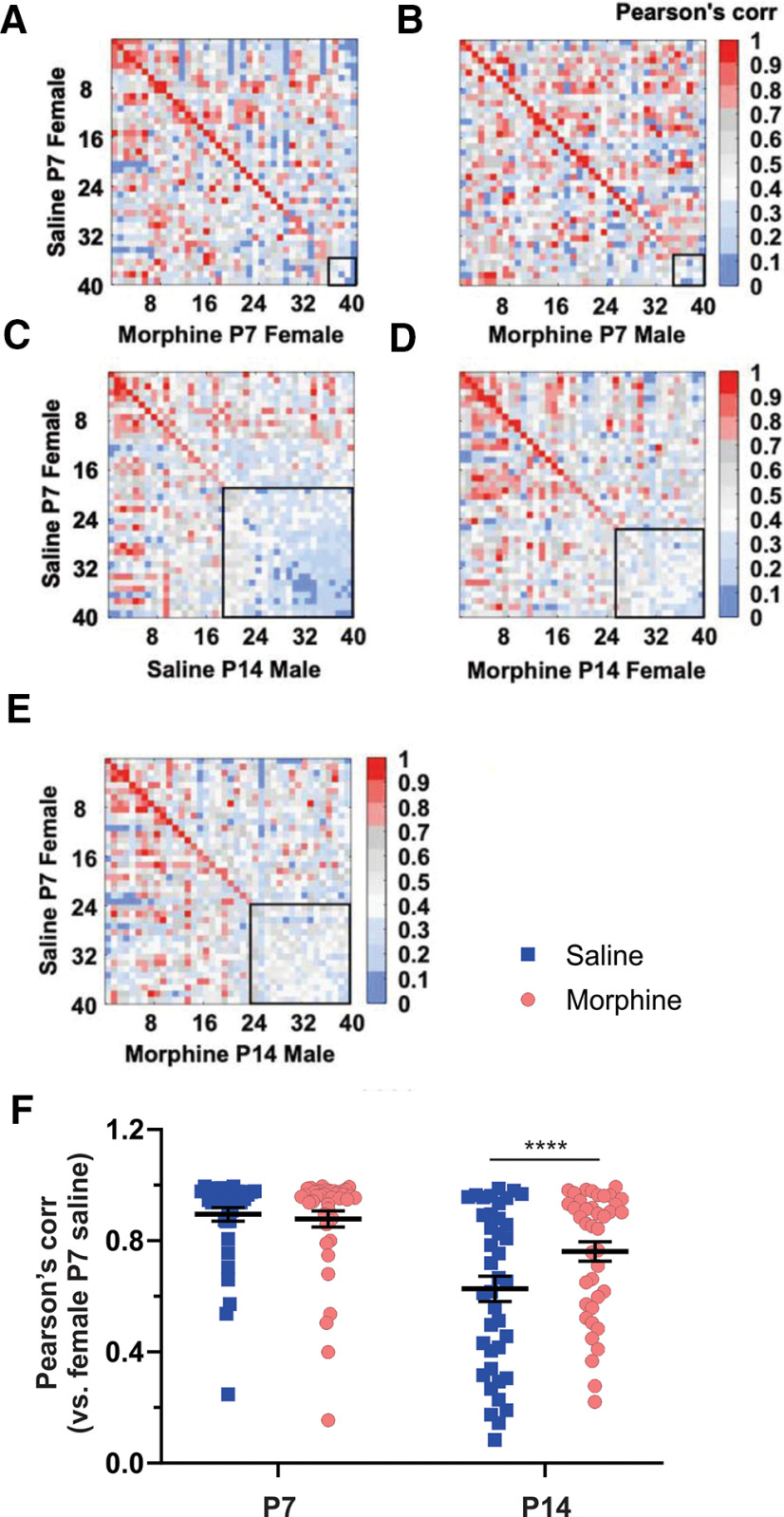
Changes in the spectrotemporal patterns of USV syllables following repeated neonatal morphine exposure at P7 and at P14. ***A–E***, Following morphine exposure: similarity matrices of the spectral types of syllable unit pairs learned from reference group (saline P7 female) compared with morphine P7 female (***A***) and male (***B***), saline P14 male (***C***), and morphine P14 female (***D***) and male (***E***). ***F***, Mean Pearson correlation values obtained from the diagonal in comparisons of all groups to the Reference Group: age: ****p <* *0.0001, *F*_(1,78)_ = 119.30; treatment: *p *=* *0.20, *F*_(1,78)_ = 1.68; postnatal day × treatment: ****p <* *0.0001, *F*_(1,78)_ = 18.17. P7 saline versus morphine, *p *=* *0.93; P14 saline versus morphine, *p =* *0.01. We performed a two-way RM ANOVA (*F*_(DFn,DFd),_ followed by a Sidak’s post-test. Data are presented as the mean ± SEM.

At P7, Pearson’s correlation values and similarity matrices indicated little effect of morphine in females or males, as demonstrated by the high correlation relative to P7 saline females (Fig. 7*A*,*B*, concentrated red hues in matrix diagonal) and by the sex-collapsed morphine group (Fig. 7*F*, left) relative to the P7 saline female reference group. These results suggest that repeated morphine administration had no detectable effect on the spectrotemporal structure of USVs on P7. Additionally, in contrast to the discordance in USV profiles noted between P7 and P14 saline mice (Figs. 6*I*,*J*, 7*C*, low correlation indicated by blue hues in matrix diagonal), morphine-treated mice showed greater USV profile similarity (or, i.e., less robust dissimilarity) at P14 when compared with the P7 saline reference group (Fig. 7*D****–****F*). Therefore, early developmental morphine exposure slowed the developmental trajectory of the USV spectrotemporal structure to more closely resemble the earlier P7 repertoire. Alternatively, the morphine spontaneous withdrawal state on P14 could mimic USV spectrotemporal effects that are consistent with a developmental delay.

### Thermal hyperalgesia following repeated neonatal morphine exposure

**Figure 8.**
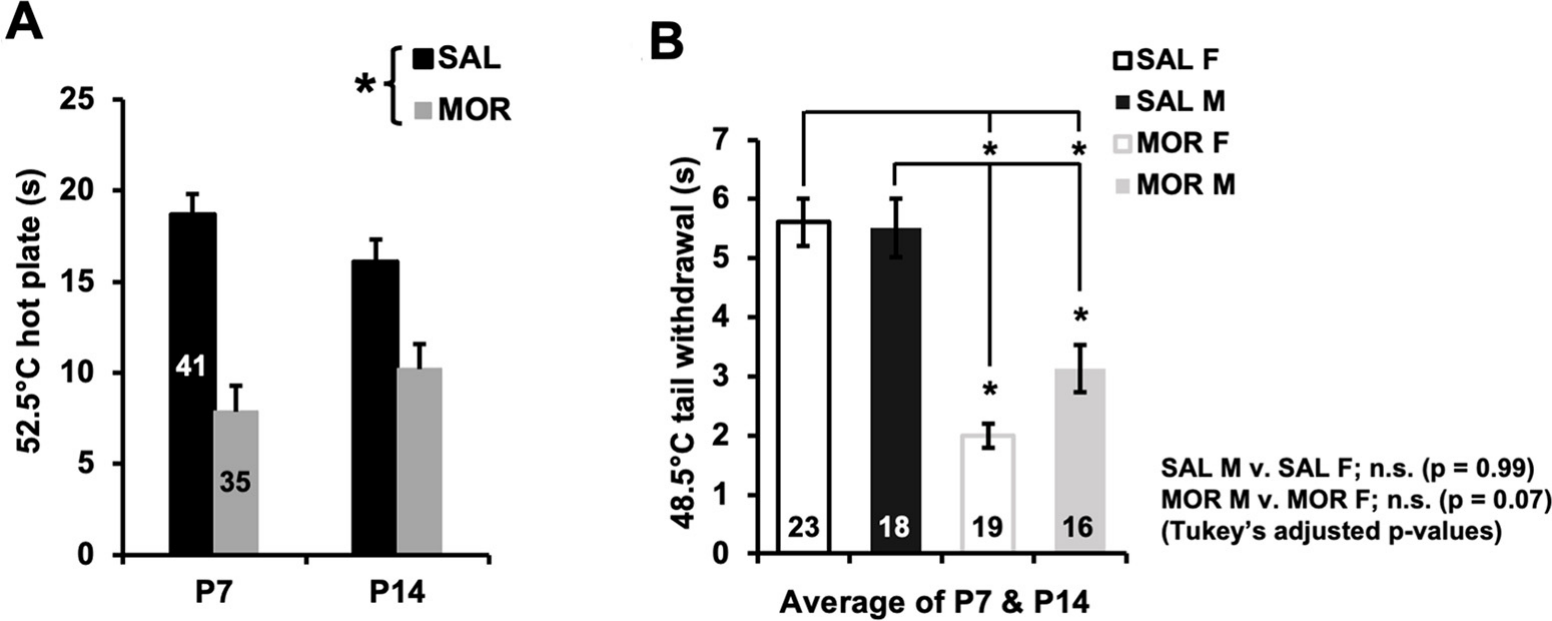
Thermal hyperalgesia following repeated neonatal morphine exposure. ***A***, In examining normalized thermal nociceptive sensitivity on the 52.5°C hot plate, RM ANOVA (treatment and sex as factors; postnatal day as the RM) revealed a main effect of treatment (*F*_(1,72)_ = 65.01, *p < 0.001) but no effect of sex (*F*_(1,72)_ = 0.52; *p* = 0.47) or postnatal day (*F*_(1,72)_ = 0.04; *p* = 0.85), and no interactions (all *p* values ≥ 0.24). Morphine-treated pups displayed a nociceptive response of 8.91 ± 1.11 s (mean ± SEM) sooner (*p* < 0.001) than saline-treated pups on average, supporting the presence of hyperalgesia. ***B***, In examining normalized thermal nociceptive sensitivity in the 48.5°C tail withdrawal assay, RM ANOVA (treatment and sex as factors, postnatal day as a repeated measure) indicated a main effect of treatment (*F*_(1,70)_ = 60.98, *p* < 0.001), postnatal day (*F*_(1,70)_ = 4.96, *p* = 0.029), and a treatment × sex interaction (*F*_(1,70)_ = 4.83, *p* = 0.03). However, there were no interactions with day (*p* values > 0.19), and, thus, tail withdrawal latencies were averaged across P7 and P14 to more clearly and specifically illustrate the treatment × sex interaction. *Post hoc* analysis revealed lower latencies in both morphine-treated males and females relative to all saline groups (Tukey’s test: SAL males vs MOR males, *p* = 0.002; SAL males vs MOR females, *p* < 0.001; SAL females vs MOR females, *p* < 0.001; SAL females vs MOR males, *p* < 0.001). While there was no difference in latencies between saline-treated males and females (*p* = 0.99), there was a nonsignificant lower latency in morphine-treated females compared with morphine-treated males (*p* = 0.072), hinting at greater hyperalgesia. Data are presented as the mean ± SEM of raw (non-normalized) data, averaged across P7 and P14.

Repeated morphine exposure from P1 to P14 led to hyperalgesia. At P7 and P14, morphine-exposed pups showed a shorter normalized latency to respond to a nociceptive stimulus relative to saline control pups on the hot plate (Fig. 8*A*) and tail withdrawal (Fig. 8*B*) assays. Morphine females tended to show enhanced hyperalgesia (shorter latencies) compared with morphine males in the tail withdrawal assay, although this result did not reach statistical significance (Fig. 8*B*; *p* = 0.07).

### Decreased locomotor activity in the open field at P21 following repeated neonatal morphine exposure

**Figure 9.**
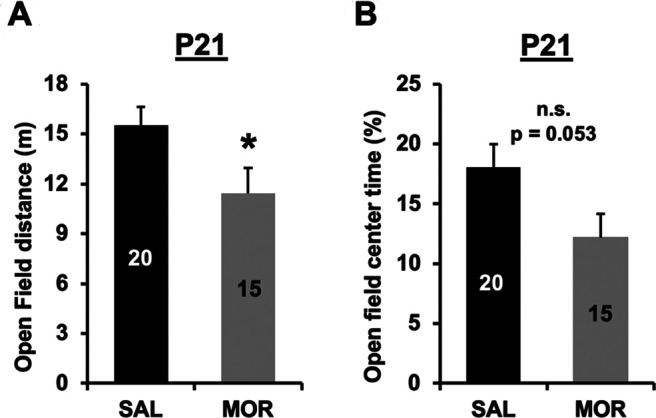
Decreased locomotor activity in the open field on P21 following repeated neonatal morphine exposure. ***A***, In examining distance traveled in the open field assay on P21, there was a main effect of treatment (*F*_(1,31)_ = 4.66, *p = 0.039), indicating that morphine-exposed mice showed less locomotor activity compared with saline control mice. There was no effect of sex (*F*_(1,31)_ = 0.07, *p* = 0.79) and no interaction (*F*_(1,31)_ = 0, *p* = 0.99). ***B***, In examining the percentage of time spent in the center of the open field, there was a nonsignificant effect of treatment (*F*_(1,31)_ = 4.15, *p* = 0.053). There was no effect of sex (*F*_(1,31)_ = 0.001; *p* = 0.97) and no treatment × sex interaction (*F*_(1,31)_ = 0.17; *p* = 0.68). We note that the decreased locomotor activity in the morphine-exposed mice could concomitantly affect the overall time spent in the center and, thus, by extension, could affect the percentage of time spent in the center of the open field as an anxiety-like measure.

We measured distance traveled and time spent in the open field arena at P21 in a subgroup of mice that were previously administered saline (*n* = 20) or morphine (*n* = 15). Prior morphine exposure was associated with decreased distance traveled compared with saline mice (Fig. 9*A*), and there was a nonsignificant decrease in the percentage of time spent in the center of the open field associated with morphine exposure (*p* = 0.053; Fig. 9*B*). Both of these effects could reflect increased anxiety-like behavior and/or decreased exploratory behavior resulting from early postnatal administration of morphine.

### Effect of spontaneous morphine withdrawal from repeated morphine exposure on P1 through P14 on the brainstem transcriptome at P15

**Figure 10.**
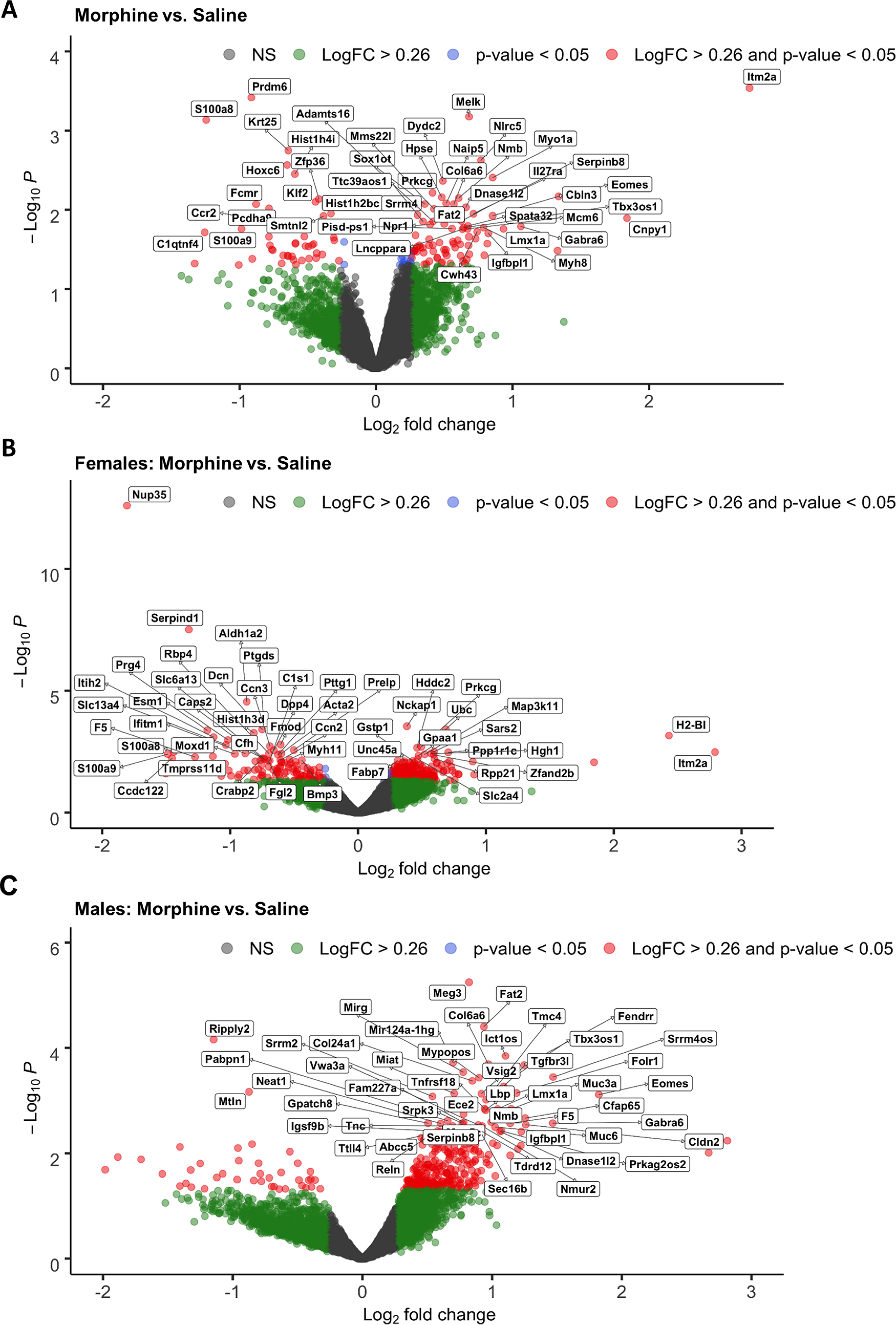
Differentially expressed genes in P15 brainstem tissue following repeated neonatal morphine exposure. ***A–C***, The effect of morphine exposure on gene expression relative to saline controls in the sex-collapsed dataset [***A***; *n* = 6 saline (3 females, 3 males), *n* = 6 morphine (3 females, 3 males)], males only (***B***; *n* = 3 saline, *n* = 3 morphine), and females only (***C***; *n* = 3 saline, *n* = 3 morphine). The *x*-axis shows gene expression as LogFC, and the *y*-axis shows Log_10_ *p* values (unadjusted). Color coding of individual genes represents LogFC > 0.26 (green), *p* < 0.05 (blue), LogFC > 0.26 and *p* < 0.05 (red), and nonsignificant (NS; gray). Complete gene lists and expression data from RNA-seq analysis are presented in Extended Data Figure 10-1 (both sexes; all genes), Extended Data Figure 10-2 (both sexes; LogFC > 0.26, *p* value < 0.05), Extended Data Figure 10-3 (females; all genes), Extended Data Figure 10-4 (females LogFC > 0.26, *p* value < 0.05); Extended Data Figure 10-5 (males; all genes), Extended Data Figure 10-6 (males; LogFC > 0.26, *p*-value < 0.05). Data from qPCR validation of differentially expressed genes (from the sex-collapsed analysis) are presented in Extended Data Figure 10-7.

We first compared morphine-exposed groups to saline control mice in the sex-combined dataset. Because mice were similarly administered twice daily morphine injections on P14 after phenotypic assessment, they were in a similar state of spontaneous morphine withdrawal on P15 when the brain tissue was harvested (10:00 A.M.; ∼16 h after the evening morphine injection on P14 at 4:00 P.M.). We identified 123 DE genes at Log_2_FC > ±0.26 and *p* < 0.05 (Fig. 10*A*, Extended Data Figs. 10-1, 10-2). Several DE genes were of interest given their potential involvement in NOWS, including the upregulation of *Slc6a2*, which encodes the norepinephrine transporter, and the downregulation of *Onecut3*, which encodes a transcription factor involved in the development of noradrenergic neurons (Espana and Clotman, 2012). We next explored the overall contribution of sex to the transcriptional changes in the brainstem induced by prior morphine exposure. In females, we identified 320 DE genes at Log_2_FC > ±0.26 and *p* < 0.05 (Fig. 10*B*, Extended Data Figs. 10-3, 10-4), including the downregulation of *Nup35*, a nucleoporin gene, and *Serpind1*, part of the serpin gene superfamily that is involved in inflammation. In males, we identified 328 DE genes at Log_2_FC > ±0.26 and *p* < 0.05 (Fig. 10*C*, Extended Data Figs. 10-5, 10-6), including *Meg3*, which encodes for a long noncoding RNA involved in brainstem development (Reddy et al., 2017). In addition, we found a unique set of DE genes in morphine-exposed males that was enriched for voltage-dependent calcium channels, including the upregulation of *Cacna1c*, *Cacna1d*, and *Cacna1e*.

10.1523/ENEURO.0143-21.2021.f10-1 Figure 10-1 Differential gene expression, sex-combined analysis. Download Figure 10-1, TXT file.

10.1523/ENEURO.0143-21.2021.f10-2 Figure 10-2 Differential gene expression, sex-combined, Log_2_FC > 0.26; *p* < 0.05. Download Figure 10-2, TXT file.

10.1523/ENEURO.0143-21.2021.f10-3 Figure 10-3 Differential gene expression, females-only analysis. Download Figure 10-3, TXT file.

10.1523/ENEURO.0143-21.2021.f10-4 Figure 10-4 Differential gene expression, females-only analysis, Log_2_FC > 0.26; *p* < 0.05. Download Figure 10-4, TXT file.

10.1523/ENEURO.0143-21.2021.f10-5 Figure 10-5 Differential gene expression, males-only. Download Figure 10-5, TXT file.

10.1523/ENEURO.0143-21.2021.f10-6 Figure 10-6 Differential gene expression, males-only, Log_2_FC > 0.26; *p* < 0.05. Download Figure 10-6, TXT file.

10.1523/ENEURO.0143-21.2021.f10-7 Figure 10-7 qPCR validation of six DE genes identified from the sex-combined RNA-seq dataset. Primer sequences for qPCR are provided, along with *p* values from the main effect of treatment from the two-way ANOVAs (sex and treatment as factors) and from the RNA-seq dataset. Download Figure 10-7, TXT file.

**Figure 11.**
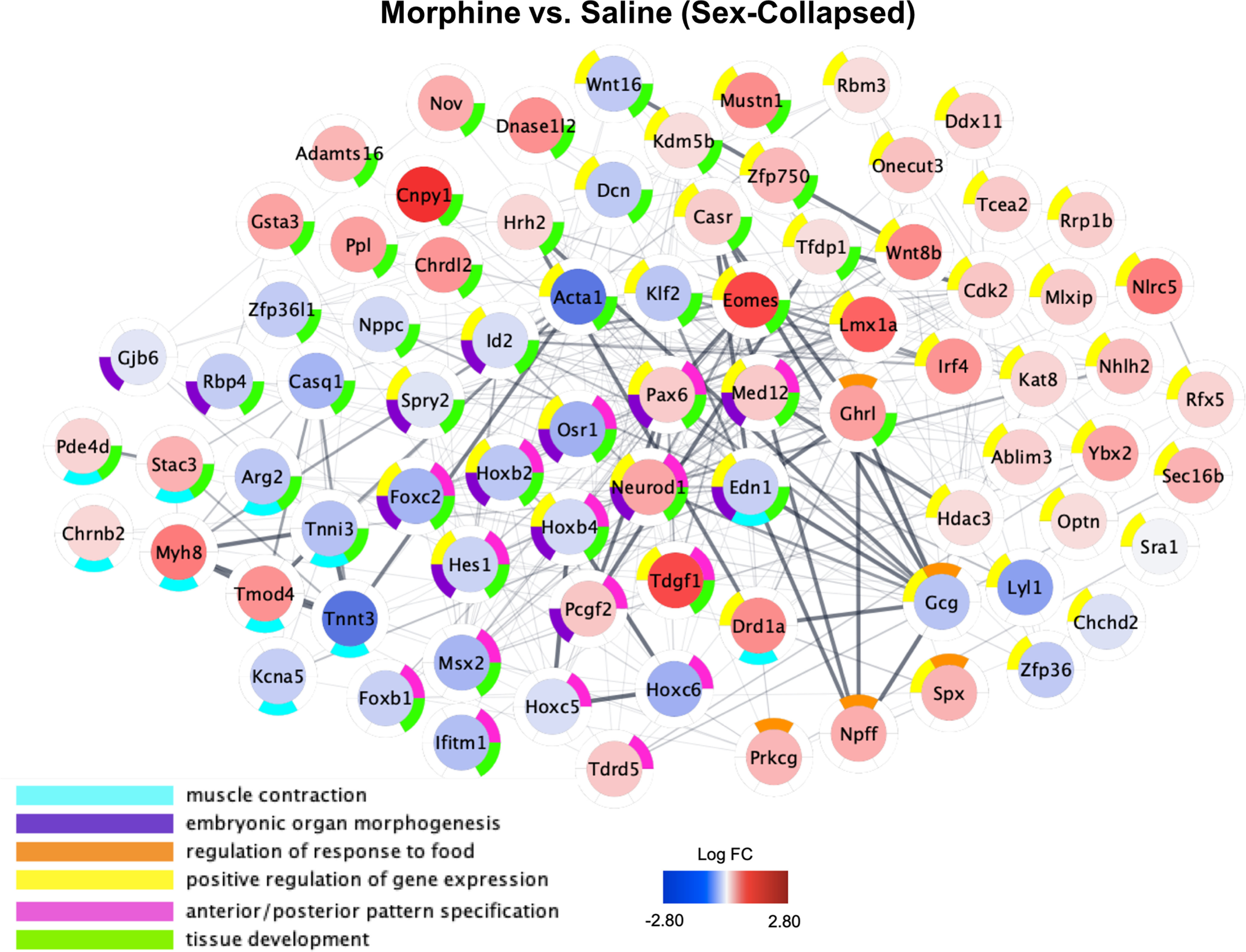
Functional network plot of differential brainstem gene expression on P15 following repeated neonatal morphine exposure. Plots represent gene expression relative to saline controls in sex-collapsed dataset [*n* = 6 saline (3 females, 3 males), *n* = 6 morphine (3 females, 3 males). Central color coding of individual genes reflects LogFC from −2.80 (blue) to 2.80 (red). Donut plots are color coded to reflect enriched GO Biological Process networks. All genes with unadjusted *p* values < 0.10 were included in functional network identification. Plots were generated using Cytoscape software, and gene interactions were imported from the STRING database to reflect known gene interactions (shown via interconnecting lines, increased line opacity represents stronger known gene interaction). Complete enrichment data for the sex-collapsed analysis are presented in Extended Data Figure 11-1.

**Figure 12.**
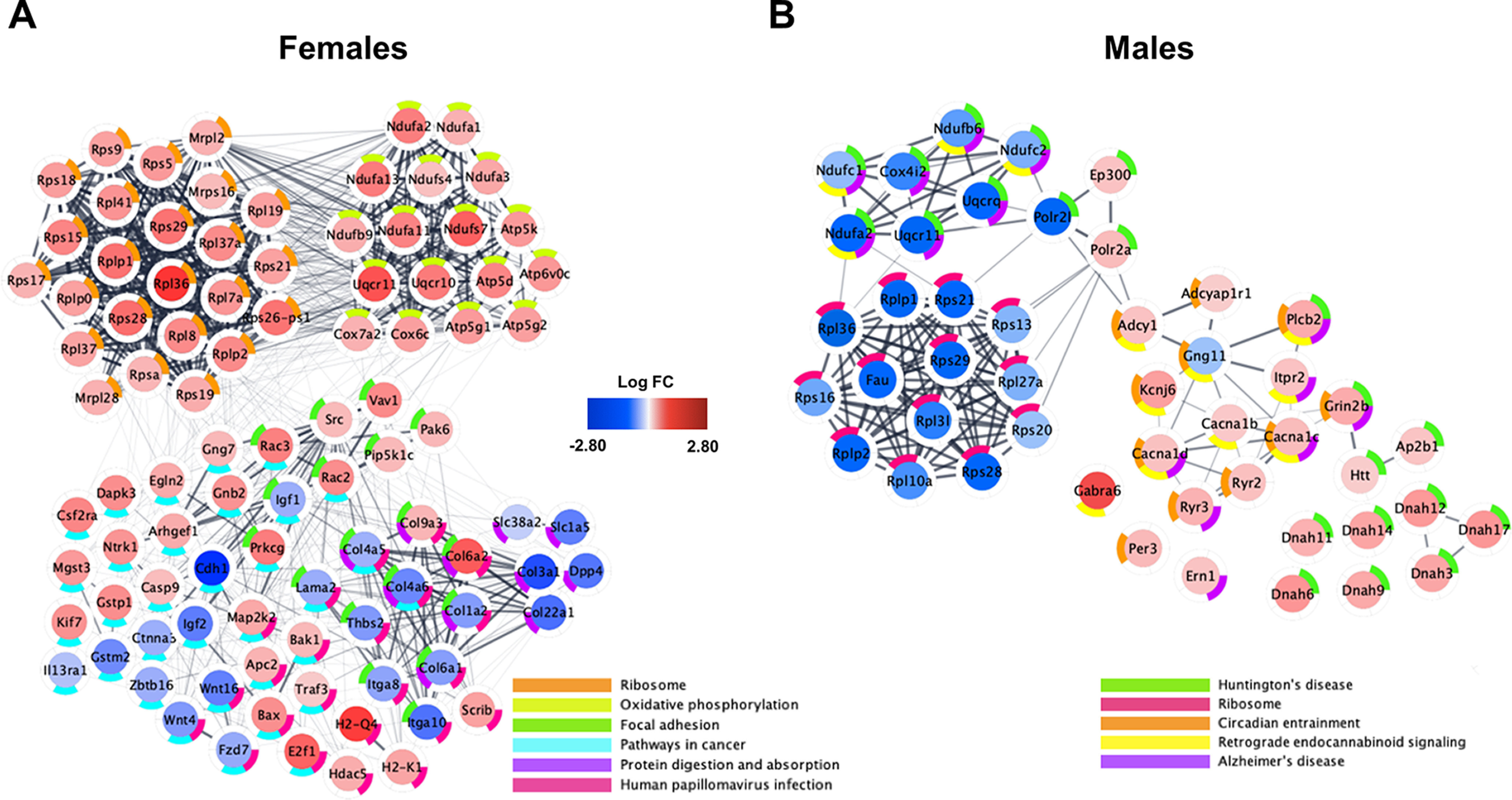
Functional network plots of differential brainstem gene expression for each sex on P15 following repeated neonatal morphine exposure. ***A***, ***B***, Plots represent gene expression relative to saline controls for females (***A***; *n* = 3 saline, 3 morphine) and males (***B***; *n* = 3 saline, 3 morphine). Central color coding of individual genes reflects LogFC from −2.80 (blue) to 2.80 (red). Donut plots are color coded to reflect KEGG enrichment pathways containing <50 implicated genes. All genes with unadjusted *p* values < 0.10 were included in functional network identification. Plots were generated using Cytoscape software, and gene interactions were imported from the STRING database to reflect known gene interactions (shown via interconnecting lines, increased line opacity represents stronger known gene interaction). Complete enrichment data are presented in Extended Data Figure 12-1 (female-only analysis) and Extended Data Figure 12-2 (male-only analysis).

We investigated the biological significance of the DE genes. For the sex-combined analysis, we identified that the top enriched pathways in morphine-exposed mice were muscle contraction, embryonic organ morphogenesis, regulation of response to food, positive regulation of gene expression, anterior/posterior pattern specification, and tissue development (adjusted *p* < 0.05; Fig. 11, Extended Data Fig. 11-1). For the females-only analysis, top enrichment terms included ribosome, oxidative phosphorylation, focal adhesion and protein digestion and absorption, pathways in cancer, and human papillomavirus infection (Fig. 12*A*, Extended Data Fig. 12-1). The top enriched pathways in morphine-exposed males were Huntington's disease, ribosome, circadian entrainment, retrograde endocannabinoid signaling, and Alzheimer’s disease.

10.1523/ENEURO.0143-21.2021.f11-1 Figure 11-1 Enrichment analysis, differential gene expression, sex-combined dataset. Download Figure 11-1, TXT file.

10.1523/ENEURO.0143-21.2021.f12-1 Figure 12-1 Enrichment analysis, differential gene expression, females-only dataset. Download Figure 12-1, TXT file.

10.1523/ENEURO.0143-21.2021.f12-2 Figure 12-2 Enrichment analysis, differential gene expression, males-only dataset. Download Figure 12-2, TXT file.

### Neonatal morphine exposure impacts alternative splicing

For all significant alternative splicing events based on differential intron/exon bin usage, see Tables 2 and 3. There were no significant alternative splicing events for the overall effect of morphine administration. However, we identified six genes (false discovery rate, <0.05), including *Ptov1*, *Ssbp4*, *Mmp2*, *D430019H16Rik*, *1700028K03Rik*, and *Lrch4*, that differed by sex and treatment (Table 1). Although there were no splicing events detected in the females-only dataset, several alternative splicing events were driven in part by the male-only morphine group that included *Ptov1*, *Brdt*, *Cdkn1c*, *Tm9sf4*, *Gipc1*, *Atg2b*, *Cul9*, and *Lrch4* (Table 2). *Gipc1* (called RGS19/GAIP-interacting protein 1) regulates μ opioid receptor agonist signaling (Wang and Traynor, 2013). For *Cdkn1c*, we detected two intronic retention events driven by the morphine males (Table 2). *Cdkn1c* (cyclin-dependent kinase inhibitor 1C) regulates the proliferation and differentiation of dopaminergic neurons in the midbrain (Andrews et al., 2007; McNamara et al., 2018). We also detected an intronic retention event within *Brdt* in males (Table 2), which codes for bromodomain testes-associated protein. The bromodomain and extraterminal family of scaffolding proteins are epigenetic readers that bind acetylated histones to recruit protein complexes and regulate chromatin dynamics and transcription, including cocaine-induced transcriptional regulation of brain-derived neurotrophic factor and neurobehavioral plasticity (Sartor et al., 2015) as well as mechanical allodynia induced by a combination of treatment with morphine and HIV glycoprotein 120 (Takahashi et al., 2018).

**Table 1:** Gene transcripts showing evidence for alternative splicing in the sex-combined dataset: morphine treatment × sex interaction

Bin_name	Symbol	Gene	Feature	Event	Locus	Locus overlap	Coordinates	Start	End	Length	Log FC	Abs LogFC	*p* value	Bin.FDR
84113:E038	84113	Ptov1	E		84,113		chr7:44863067–44869788	44867455	44867512	58	−2.76	2.76	2.87E-07	0.025
76900:E029	76900	Ssbp4	E		76,900		chr8:70597490–70608872	70599525	70599537	13	−2.93	2.93	3.96E-07	0.025
17390:I024	17390	Mmp2	I		17,390		chr8:92827291–92853420	28546290	28598105	51816	−1.28	1.28	4.46E-07	0.025
268595:I001	268595	D430019H16Rik	I		268,595		chr12:105453856–105493095	78291839	78291961	123	7.63	7.63	1.04E-06	0.044
76421:I007	76421	1700028K03Rik	I		76,421	231,570	chr5:107507621–107580596	1.33E + 08	1.33E + 08	484	−8.26	8.26	1.34E-06	0.045
231798:E017	231798	Lrch4	E		231,798	1E + 08	chr5:137629121–137641099	1.38E + 08	1.38E + 08	2	−4.87	4.87	1.75E-06	0.049

Abs = absolute.

**Table 2:** Gene transcripts showing evidence for alternative splicing in the males-only dataset: effect of morphine treatment

Symbol	Gene	Feature	Event	Locus	Locus overlap	Gene coordinates	Start	End	Length	LogFC	*p* Value	bin.FDR
114642	Brdt	I		114,642		chr5:107331159–107387058	1.42E + 08	1.42E + 08	27158	−1.38124	2.70E-08	0.003
12577	Cdkn1c	I		12,577		chr7:143458339–143461050	1.43E + 08	1.43E + 08	3842	−1.32251	5.20E-08	0.003
12577	Cdkn1c	I		12,577		chr7:143458339-143461050	1.43E + 08	1.43E + 08	5304	−1.3707	1.25E-07	0.0047
84113	Ptov1	E		84,113		chr7:44863067-44869788	44867455	44867512	58	1.98688	1.38E-07	0.0047
99237	Tm9sf4	I		99,237		chr2:153161303–153210466	73861694	73862277	584	−2.91739	2.33E-07	0.0066
67903	Gipc1	I		67,903		chr8:83652677–83664694	30900397	30900637	241	−1.22149	1.83E-06	0.041
76559	Atg2b	I		76,559		chr12:105616136–105685211	30121066	30121475	410	3.349581	1.93E-06	0.041
78309	Cul9	E		78,309		chr17:46500605–46546388	46521041	46521156	116	2.133526	2.59E-06	0.049
231798	Lrch4	E		231,798	1E + 08	chr5:137629121-137641099	1.38E + 08	1.38E + 08	2	3.464074	2.90E-06	0.0495

**Table 3:** Differentially expressed gene transcripts (*p* < 0.05) that significantly correlated (*p* < 0.05) with NOWS-model phenotypes on P14

Phenotypes	Overlapping phenotypes, *n*	Genes, *n*	Differentially expressed genes (MOR) correlated with phenotypic data
Hot plate latency; normalized body weight; USVs per minute; USV distance	4	1	*Prokr1*
Hot plate latency; normalized body weight; USVs per minute	3	3	*Smtnl2*, *Alox5ap*, *Adamts16*
Normalized body weight; USVs per minute	2	15	*Hist1 h2bc*, *Id2*, *Spata32*, *Krt25*, *Hist3 h2a*, *Cox7b2*, *Zmym3*, *Naa40*, *Col6a6*, *Srrm4*, *Emcn*, *Hpse*, *Miat*, *Mapk1ip1*, *Lncppara*
Normalized body weight; hot plate latency	2	8	*Nlrc5*, *Wnt8b*, *Tbx3os1*, *Pla2g4b*, *Fcmr*, *Prickle3*, *Cdca5*, *Prdm6*
Normalized body weight; tail withdrawal latency	2	4	*Myo1a*, *Plpp5*, *Mir3060*, *Mis18a*
Tail withdrawal latency; USVs per minute	2	1	*Magel2*
Tail Withdrawal Latency	1	19	*Selenov*, *Myom3*, *Ebna1bp2*, *Il27ra*, *Fam92b*, *Ccdc116*, *Vipr1*, *Ccr2*, *Prkcg*, *Rsg1*, *Pisd-ps1*, *Pisd*, *Arg2*, *Mfsd11*, *Mfap5*, *Msr1*, *Ifi207*, *Foxc2*, *Fggy*
Normalized body weight	1	14	*Tcea2*, *Mtln*, *Thsd7b*, *Snapc1*, *Klf2*, *Mirg*, *Galnt15*, *Ccdc150*, *Cdk2*, *Mms22l*, *Melk*, *Ccdc15*, *Tdgf1*, *Hoxc6*
USVs per minute	1	11	*Timp1*, *Chrdl2*, *Cldn11*, *Rfc5*, *Plekhg4*, *Myh8*, *Snrnp200*, *Cpsf4l*, *Gng11*, *Wdr66*, *Fat2*

### Correlations of DE genes on P15 with NOWS behavioral phenotypes displayed on P14

Notably, 75 of 139 DE genes (∼54%) correlated with at least a single behavioral phenotype at P14, and 32 of 139 DE genes (∼23%) correlated with at least two phenotypes (Table 3). Interestingly, the expression of *Prokr1* was significantly correlated with the most phenotypes, including body weight (*R* = −0.81, *p* = 0.001), hot plate latency (*R* = −0.58, *p* = 0.04), total number of USVs (*R* = 0.65, *p* = 0.02), and distance traveled during USV recording session (*R* = 0.58, *p* = 0.04). In morphine-exposed mice, *Prokr1* was upregulated compared with saline mice, and expression was negatively correlated with hot plate latency. Thus, upregulation of *Prokr1* at P15 was associated with heightened behavioral indications of hyperalgesia at P14. Interestingly, Prokr1 (prokineticin receptor 1) codes for a GPCR for the chemokine prokineticin and is upregulated in nociceptors during tissue inflammation and contributes to inflammatory pain (Franchi et al., 2017). Thus, upregulation of *Prokr1* could potentially be a critical mediator of sensory, motivational, and affective model behaviors following and during neonatal opioid withdrawal.

## Discussion

We administered morphine from P1 to P14 in mouse pups, a developmental period approximating the third trimester in human neonates, to model NOWS. We found several important NOWS-related phenotypes in female and male mice, including reduced weight gain, developmental motor delays and USVs, agitation, and decreased locomotor activity in the open field test of anxiety-like behavior, and thermal hyperalgesia. We also discovered potential sex-specific vulnerabilities to NOWS model behaviors. In human neonates, males are more frequently diagnosed with and treated for NOWS, with no overall increase in NOWS severity compared with females (Juni et al., 2008). Increased male sensitivity to NOWS has also been found in C57BL/6J male mice, as evident by an increase in USVs (Robinson et al., 2020). However, our findings suggest that outbred CFW female mice were more sensitive to the NOWS neurobehavioral phenotypes than males (e.g., delayed latencies to self-right and increased USVs on P7), suggesting sex-specific neurodevelopmental delays in NOWS. Motor delays and an earlier emergence of increased USVs were also reported in C57BL/6J female mice (Robinson et al., 2020). Thus, repeated bouts of MOR intoxication and withdrawal during the third trimester-equivalent exposure is sufficient to induce multiple signs of neurodevelopmental delay. Overall, female mice were more vulnerable to the effects of morphine exposure during P1–P14. Importantly, we also found sex-specific transcriptional alterations in the brainstem following morphine administration, with enrichment of ribosomal and oxidative phosphorylation pathways for both sexes. Collectively, our findings begin to highlight the consequences of morphine exposure during a critical neurodevelopmental period.

Emission of USVs by pups is exhibited during distress (Hofer, 1996), and they typically peak at P7 and then decrease significantly by P14 (Elwood and Keeling, 1982). Consistent with previous studies in rats (Barr and Wang, 1992; Barr et al., 1998; Jones and Barr, 2001; Jones et al., 2002; Kalinichev and Holtzman, 2003) and mice (Robinson et al., 2020), we observed a decrease in USVs from P7 to P14 in saline control mice and, to some extent, in morphine-exposed mice, but the decrease was less pronounced in morphine-exposed mice, as indicated by a significantly greater number of USVs on P14 compared with saline control mice. The reinforcement of USVs during maternal separation may be mediated endogenous opioids (Hamed et al., 2012) as μ opioid receptors are required for this behavior (Moles et al., 2004). Thus, adaptations in endogenous opioid signaling in morphine-exposed pups could contribute to the increase in USVs on P14. Changes in USVs emerged earlier in morphine-exposed female mice compared with male mice during opioid withdrawal. An earlier emergence of augmented USVs in female mice contrasts with prior studies using NOWS-related models where C57BL/6J inbred males were more vulnerable to consequences of morphine on USVs (Robinson et al., 2020). Inconsistencies between these studies could be because of several factors include morphine dose, frequency and interval of morphine administration, and the time course of behavioral analysis. Another factor could be genetic background because we tested outbred CFW mice whereas a recent report that used a similar protocol used C57BL/6J mice (Robinson et al., 2020). Given our results, future studies using multiple genetic backgrounds of mice, including not only diversity outbred mice (Saul et al., 2019), but also reduced complexity crosses (Bryant et al., 2018, 2020) would be valuable for further investigating the genetic, molecular, and physiological contributions to the development of NOWS and the long-term consequences.

We found sex-specific effects on thermal nociception in our NOWS mouse model. Previous studies reported withdrawal-induced hyperalgesia and allodynia in rat pups following early neonatal opioid exposure, including increased thermal nociception (Sweitzer et al., 2004; Zhang and Sweitzer, 2008), increased formalin-induced inflammatory nociception in P11 rats (Zissen et al., 2006), and mechanical allodynia (Sweitzer et al., 2004; Zissen et al., 2007; Zhang and Sweitzer, 2008). Here, we showed that early neonatal morphine exposure was sufficient to induce hyperalgesia. Notably, this hyperalgesia emerged earlier in morphine-exposed females. Our findings support previous work in adult outbred CD-1 female mice that showed an earlier emergence and more persistent augmentation in opioid-induced hyperalgesia (Holtman and Wala, 2005; Juni et al., 2010; Arout et al., 2015). Enhanced thermal hyperalgesia and mechanical allodynia were also observed in female rats during spontaneous opioid withdrawal, although it is unknown whether similar mechanisms in the brain lead to hyperalgesia in neonatal versus adult females. Evidence suggests that neuroadaptations of NMDA receptor signaling in males may protect against the early emergence of developmental delays. Interestingly, *Grin2b*, the gene coding for the GluN2B subunit of the NMDA receptor, was significantly upregulated only in morphine-exposed male mice, consistent with male-specific morphine-induced neuroadaptations of the NMDA receptor system (Charles et al., 2017). Despite keeping the pups warm between behavioral testing using an electric heating pad, one limitation of this study is that we did not monitor the body temperature of the neonates throughout testing. Because hypothermia in neonatal body temperature is known to induce USVs from the pups (Branchi et al., 1998), and because opioid withdrawal in mice (Belknap, 1989) and neonatal rats (Thornton and Smith, 1998) has been shown to be associated with hypothermia, it is possible that the detection of the effect of prior morphine exposure on USV phenotypes depends on the presence of withdrawal-induced hypothermia to evoke USV responses.

To explore potential mechanisms that contribute to sex-specific effects of early neonatal morphine on behavior, we completed RNA-seq on the brainstems of female and male mice at P15, ∼16 h following their final morphine injection. We identified distinct gene sets and enriched pathways between morphine-exposed male and female mice. In females, highly significant enrichment terms were identified for 25 ribosomal genes and several genes coding for mitochondrial proteins and oxidative phosphorylation, all of which were upregulated in response to morphine. We identified the retinaldehyde dehydrogenase, *Aldh1a2*, as a significantly downregulated gene in morphine-exposed females. ALDH1A2 synthesizes retinoic acid during early stages of development and has been linked to neural tube defects (Niederreither et al., 2002). Another gene involved in neurogenesis and cortical development, *Cdh1* (Delgado-Esteban et al., 2013), was also reduced in the brainstems of morphine-exposed female mice, suggesting that morphine exposure and spontaneous withdrawal from morphine may disrupt neurodevelopmental processes by altering the expression of key genes. In addition, we identified the upregulation of genes involved in nociception, including *Pnoc*, a gene coding for the precursor protein to nociceptin. Nociceptin levels in the spinal cord were higher in female rats treated with morphine (Zhang et al., 2012). Together, our findings support the notion that early postnatal exposure to morphine leads to changes in nociceptin signaling, although the potential role in hyperalgesia during opioid withdrawal in neonates is unknown.

The multiple correlations that we identified between normalized read counts for the DE gene Prokr1 and behavioral phenotypes, including hot plate hyperalgesia (negative correlation with latency), USV frequency, and distance traveled during USV recordings, and its known role in inflammation and pain (Franchi et al., 2017) make this a compelling candidate mechanistic component of nociceptive, motivational, and affective components of pain during spontaneous opioid withdrawal in neonatal mice that warrants further study.

Enrichment of pathways in morphine-exposed male mice were largely distinct from those of morphine-exposed female mice. We observed several pathways enriched in male mice following morphine exposure, including circadian entrainment. Within the circadian entrainment pathway, the following genes were significantly upregulated in morphine-exposed male mice: *Per3* (circadian gene); *Adcyap1r1* (PACAP1 receptor); *Ryr2,3* (intracellular calcium channels); *Cacna1c,d,e* (membrane voltage-gated calcium channels); *Grin2b* (NMDA receptor subunit); *Kcnj6* (Kir3.2/GIRK2 potassium channel); and *Kcnj13* (Kir7.1 potassium channel). Chronic morphine administration altered the expression of calcium and potassium channels in the mouse cortex and limbic system that contributes to opioid withdrawal, tolerance, and hyperalgesia (Shibasaki et al., 2007, 2010, 2011; Cruz et al., 2008; Lee et al., 2011). Importantly, circadian genes, such as *Per3*, which was changed in morphine-exposed male mice, have been associated with opioid-induced hyperalgesia in mice (Zhang et al., 2019) and mutations in *PER3* are associated with opioid dependence in humans (Surovtseva et al., 2012). Other genes that were upregulated in morphine-exposed males include the PACAP1 receptor and ryanodine receptors, which are also associated with opioid withdrawal (Ohsawa and Kamei, 1999; Mácsai et al., 2002; Martin et al., 2003; Lipták et al., 2012).

In both females and males, we found an upregulation of *Slc6a2*, which encodes for the norepinephrine transporter. This transcriptional adaptation might serve to counter the increase in extracellular norepinephrine levels, a hallmark feature of opioid withdrawal (Koob, 2020). Indeed, it is well established that reducing norepinephrine release by pharmacological agonism at the α2 receptor with clonidine can alleviate the severity of the sympathetic component of opioid withdrawal (Gowing et al., 2016). Our findings suggest that clonidine might also be an effective treatment for autonomic symptoms in NOWS. Unfortunately, we were unable to validate the RNA-seq results via real-time quantitative PCR (Extended Data Fig. 10-7). Discordance between RNA-seq and quantitative PCR (qPCR) data are not uncommon and could be attributed not just to our limited sample size, but also to the normalization method used for RNA-seq analysis, the exons we chose to target with qPCR compared with gene-level output from RNA-seq, and/or the disparate sensitivity of these two methods of quantification (Rajkumar et al., 2015; Everaert et al., 2017). We acknowledge that our sample size for analysis with RNA-seq is small (*n* = 6 SAL, *n* = 6 MOR), and caution against any major conclusions before there are more in-depth studies and replication in CFW mice.

Human neonates born with NOWS often have a lower birth weight and have difficulty with postpartum weight gain. Consistent with other mouse models of NOWS (Robinson et al., 2020), we found a significant reduction in weight gain in morphine-exposed pups that persisted from P14 through weaning into adulthood. Reduced weight gain can be caused by several factors including the compromised ability to feed or reduced motivation to feed. Opioids inhibit gastrointestinal motility, inducing nausea and leading to anorexia (Santolaria-Fernández et al., 1995). Opioid-induced weight loss exerts profound effects on nutrition, health, and physiology in OUD (Santolaria-Fernández et al., 1995) and may be a hallmark in the transition to opioid dependence (Chen et al., 2006).

Collectively, we demonstrated sex-specific phenotypes using a NOWS model in outbred mice and revealed potential brainstem mechanisms of opioid withdrawal severity related to neurodevelopmental delays. Our studies serve as the foundation for future endeavors to examine the transcriptional changes in additional brain regions and specific cell types related to NOWS-related behavioral phenotypes. Our work establishes divergent neurobiological adaptations between females and males that could have implications for treatment and prevention of NOWS. Finally, because the sex differences we have identified contrast with previous reports on a different genetic background (Robinson et al., 2020), they suggest a potential genetic component that should be further explored on multiple genetic backgrounds.

## Synthesis

Reviewing Editor: Karen Szumlinski, University of California at Santa Barbara Decisions are customarily a result of the Reviewing Editor and the peer reviewers coming together and discussing their recommendations until a consensus is reached. When revisions are invited, a fact-based synthesis statement explaining their decision and outlining what is needed to prepare a revision will be listed below. The following reviewer(s) agreed to reveal their identity: Julie Blendy, Susan Maloney.

Upon consultation with two experts reviews, we have arrived at the decision of Revise-Re-review. Both reviewers were very enthusiastic over the focus on developing an outbred mouse model of neonatal opioid exposure and we all agree that the report is timely. The decision to request a revision in based on some common concerns related to the interpretation of the data vis-a-vis the timing of euthanasia (are the authors modeling withdrawal or early opioid exposure?), issues related to the statistical analyses and confusion regarding figure labeling, call-outs and order in the uploaded manuscript. In addition to these common concerns, both reviewers offered additional suggestions for revision that should help clarify the specific procedures and data interpretation. These requested revisions are likely to take fewer than 2 months to address; however, should you require more time to complete the revision process due to pandemic-related complications, please feel free to reach out to the eNeuro office and request an extension.

Please find the specific comments of the reviewers below:

Reviewer 1:

The manuscript eN-NWR-0143-21 describes the impact of neonatal opioid exposure in an outbred mouse strain. The paper covers phenotypes ranging from USVs, pain sensitivity and anxiety-like behavior and attempts to correlate some of these with gene expression data from the midbrain structures. The choice of an outbred strain is commendable as diversity in genetic background is a factor that many investigators are just now beginning to appreciate. Further, this strain would, as the authors state, be used for subsequent studies for genetic mapping.

Given the increase in opioid use and the inadvertent effects on newborns, the studies are timely. While there are some interesting aspects of the study overall, there are several questions and concerns relating to the design and results that are outlined below:

The issue of when behaviors vs molecular endpoints were measured is unclear. The timing is important as it will established if mice are in withdrawal or under the influence of morphine when behaviors are assessed. The authors state that mice are injected at 0730 and 1600 daily.

Only on P14 do that state injections were done at 1000 and 1600 so if the behavior assessments on P 14 were done between 0800 -1000 then mice are in WD (if last injection was at 1600 the day before, if after 1000 then mice are under the influence of morphine. And what was the case when behaviors were assessed on P7? Gene expression analysis was done on P15, well into the WD state. Thus, clarification is needed here. If behavior and gene expression was collected in a WD state, the authors should state this, if not, the correlations between behavior and gene expression are meaningless.

A large portion of the analysis is focuses on USVs. The authors should comment on the significance of the changes they see. Rather than simply stating that “repertoires are developmentally regulated” it would be beneficial for the reader to know what is expected in +/+ mice vs what is seen in opioid exposed mice. Do USVs increase over time and this is exacerbated in opioid exposed mice or do they decrease over time and opioid exposure keeps them from decreasing.

Fig 4 is very unclear in that the y-axis has USV total distance, or distance/min. What exactly is being quantified here? The figure does not seem to fit with the legend.

I was very confused by the figure legend vs figures for Fig 7. The legend describes locomotor activity, but the figure portrays a number of Pearson’s’ correlation plots. There is also no reference to Fig. 7 in the text of the manuscript. With respect to the Pearson’s correlation s., I really don’t understand what information the correlations will provide comparing Saline P7 and morphine P14. Or saline P7 females and morphine P14 male??

The RNA seq analysis is a nice addition, however the fact that all genes with an unadjusted <0.10 were used for analysis is curious. P<0.05 is typically used. Can the authors justify?

The paper would have been greatly strengthened by a few validations of genes that were highlighted in the discussion. These genes could have major implications in our understanding of what is happening at a molecular level following neonatal opioid exposure and withdrawal, but the fact that nominal P values were used combined with lack of validation lessens the impact of this data.

Reviewer 2:

Overall, this study is an examination of the effects of early-life opioid exposure in the mouse, as a model of neonatal opioid withdrawal syndrome (NOWS) on neurodevelopmental outcomes including USVs, pain reflexes, and later anxiety-related behavior, as well as an investigation into the transcriptomic consequences of this exposure. Few models exist to aid our study of the neurobiological impact of early-life opioid exposure. This study presents a new model, and provides neurobehavioral benchmarks of opioid exposure and their transcriptomic correlates.

Specific comments:

Methods:

1. Did the power calculations described in the methods include sex as a factor such that 6/sex/Tx is sufficient to observe any sex x drug Tx interactions?

2. Were the randomly assigned saline-tx pups within-litter controls? Or did all pups in a litter receive the same drug exposure? Clarity in the methods would be helpful to understand if litter effects or litter size need may be a concern.

3. Previous research has demonstrated that sex of the experimenter can influence behavioral performance of rodents in a sex-dependent manner (e.g. Sorge et al, Nature Methods, 2014; Bohlen et al, Behav Brain Res, 2014). Reporting the sex of the experimenter here would aid in reproducibility and comparisons with other models and work in this area.

4. Was the temperature of the pups collected at the time of USV recording? Temperature is known to increase USV production - a cold pup will produce more calls to elicit retrieval and care from the dam (Branchi et al., 1998, PMID 9810475). It would be helpful to show here if the increase in call rate in the morphine-treated pups was secondary to possible temperature dysregulation.

5. Clarity is needed in the statistical section. The fit of the distributions with the assumptions of univariate analysis needs to be reported to support the use of the parametric testing in the absence of transformations. In addition, the term “mixed” ANOVA was used indicating random and fixed effects were included in the model. If a true mixed ANOVA was used, please indicate which factors were treated as random. Figure 1 legend suggests that Treatment was a random factor - and while this was randomly assigned, in the statistical model it is likely more appropriate to treat it as a fixed effect since those are the variables within a model that are expected to influence the outcome measure and random effects are those for which the variance attributed may need to be controlled (like batch effects or litter effects) but that aren’t expected to impact the outcome.

Results:

1. Were there any deaths in the saline-treated group, suggesting deaths may be attributable to injection issues? If not, please justify the morphine dose used here. Is it translationally relevant? The pup deaths suggest it may be on the higher end? Are pup deaths typical at this dose at these ages?

2. No significant sex x treatment or sex x treatment x day interactions were reported in the Fig 1 legend. For clarity, it would be helpful to add a statement to the results indicating sex did not interact with treatment to influence weight gain.

3. For the righting reflex data, is there justification for examining sex x treatment effects on P7 independently or within sex & treatment comparisons across ages since the three-way interaction of sex x treatment x day was not statistically significant in the omnibus model? If these were post hoc analyses, was a correction applied for multiple comparisons? In addition, the variance within the females (and the saline-treated groups on P4) seems large. Was there homogenous variance between groups? If not, might a non-parametric or transformation be appropriate?

4. In lines 249-250, the sentence describing the lack of sex effects within each treatment group across time on USVs on P14 reads like there is no difference between groups with the wording “compared to the saline group”. Rewording this sentence would help clarify that the treatment effect was strong and sustained, but there was no interaction with sex at this age.

5. For the USV data in Figure panel 3B, what is the justification for examining Treatment x Sex x Minute effects without a significant 3-way interaction?

6. For USV syllable repertoire data in Figure 4, in the legend it is stated that there were significant effects of Development x Group Factors for 5/6 outcomes. For Frequency Bandwidth, what is the justification for examining within Development (which we assume is age here?) differences across sex & treatment since the interaction was not significant? What is “Group Factors” here. Does that mean a predictor with 4 levels - F Mor, M Mor, F Sal, and M Sal? Why not add sex and treatment as their own factors in this model? That might provide interesting interactions between sex and treatment that cannot be observed with a 4-level factor. The use of the term “sex-specific differences” in line 261 doesn’t seem appropriate without sex x treatment analysis.

7. The correlation of syllable types between groups to show sex differences is a really interesting and informative way to approach these data.

8. For the correlation data in Fig 6G, why was a one-way ANOVA used over a three-way ANOVA with sex, treatment and age as factors? If these are the same mice recorded at both P7 and P14, then the data are likely not independent and a within-subjects factor may be necessary.

9. The ANOVA statistics should be included in the Figure legends 6G and 5J.

10. For the open field data, why was an Unpaired t-test conducted between the treatment groups for percent time in center when the Treatment effect in the ANOVA was significant and already revealed this effect?

11. The interpretation of the open field decreased percent time in center by the MOR animals as increased anxiety-related behavior is confounded by the overall hypoactivity. Because the mice traveled less around the arena, as a result they are in the center area less - even showing these data as percent of total time in the center. I think the interpretation here needs to be coupled with acknowledgement of the confound.

Minor comments:

1. The word “Opioid” is missing from the NOWS definition in line 31.

2. The word “during” is missing from line 99 before “behavioral testing”.

3. Line 228 uses the word “lead” instead of “led”.

4. Increasing the font size of the syllable plots in Figure 5 would increase legibility.

5. Line 266 - is this Fig 6C of 5C?

6. The figure number citations appear to be mixed up between the number they are given in the manuscript, cited in the text and labeled in the legends.

7. The panels for Fig 8 are not described in the correct order - B is not described and C is labeled as B.

8. A word is missing from line 375 “then by P14”.

9. Line 369 is missing “by”.

## Author Response

Reviewer 1:

The manuscript eN-NWR-0143-21 describes the impact of neonatal opioid exposure in an outbred mouse strain. The paper covers phenotypes ranging from USVs, pain sensitivity and anxiety-like behavior and attempts to correlate some of these with gene expression data from the midbrain structures. The choice of an outbred strain is commendable as diversity in genetic background is a factor that many investigators are just now beginning to appreciate. Further, this strain would, as the authors state, be used for subsequent studies for genetic mapping.

Given the increase in opioid use and the inadvertent effects on newborns, the studies are timely. While there are some interesting aspects of the study overall, there are several questions and concerns relating to the design and results that are outlined below:

The issue of when behaviors vs molecular endpoints were measured is unclear. The timing is important as it will established if mice are in withdrawal or under the influence of morphine when behaviors are assessed. The authors state that mice are injected at 0730 and 1600 daily.

Only on P14 do that state injections were done at 1000 and 1600 so if the behavior assessments on P 14 were done between 0800 -1000 then mice are in WD (if last injection was at 1600 the day before, if after 1000 then mice are under the influence of morphine. And what was the case when behaviors were assessed on P7? Gene expression analysis was done on P15, well into the WD state. Thus, clarification is needed here. If behavior and gene expression was collected in a WD state, the authors should state this, if not, the correlations between behavior and gene expression are meaningless.

RE: We apologize for the lack of clarification. Behavior was analyzed during spontaneous opioid withdrawal on P7 and P14. The timing of the injections was switched from 0730/1600 to 1000/1600 on P7 and P14 to allow time for phenotyping between 0800 and 1000. Because mice received twice daily morphine injections on P14 to maintain a state of dependence, the mice were in a similar state of spontaneous withdrawal when they were sacrificed on P15 (approximately 16 h after the final morphine injection on the evening of P14).

We provide this text in the Methods to clarify:

"For P1 through P14, injections were administered twice daily mostly at 0730 h and 1600 h. The only exceptions were P7 and P14 in which injections were administered at 1000 and 1600 h to provide time for phenotypic assessment of spontaneous morphine withdrawal from 0800-1000 (see below).”

We have altered the sub-header in the Results to further clarify this point and added additional introductory text:

"Effect of spontaneous morphine withdrawal on the brainstem transcriptome at P15"

We first compared morphine-exposed groups to saline control mice in the sex-combined dataset. Because mice were similarly administered twice daily morphine injections on P14 after phenotypic assessment, they were in a similar state of spontaneous morphine withdrawal on P15 when the brain tissue was harvested (1000 h; approximately 16 h after the evening morphine injection on P14).

A large portion of the analysis is focuses on USVs. The authors should comment on the significance of the changes they see. Rather than simply stating that “repertoires are developmentally regulated” it would be beneficial for the reader to know what is expected in +/+ mice vs what is seen in opioid exposed mice. Do USVs increase over time and this is exacerbated in opioid exposed mice or do they decrease over time and opioid exposure keeps them from decreasing.

RE: We have added text stating that in unexposed mice, USVs normally decrease over time from P7 to P14 as the pups develop. The fact that we do not see this same decrease from P7 to P14 in the mice with repeated morphine exposure suggests that there could be a developmental delay as a result of treatment (i.e., ’prior opioid exposure keeps them from decreasing’ as they would in the normal developmental trajectory). The alternative explanation is that the state of morphine withdrawal, rather than a developmental delay, is responsible for the greater number of USVs on P14 and thus, the state of morphine withdrawal “mimics” the less developed P7 state.

End of Results section for increased USVs:

"As expected, control pups receiving saline displayed an overall developmental decrease in USVs from P7 through P14 (Noirot, 1966) (Fig.3A). Notably, morphine exposed pups emitted more USVs compared to saline pups on P14, but not on P7 (Fig.3A) which could either indicate a developmental delay or could be explained by the spontaneous morphine withdrawal state.”

End of Results section on syllables:

"Therefore, early developmental morphine exposure slowed the developmental trajectory of the USV spectrotemporal structure to more closely resemble the earlier P7 repertoire. Alternatively, the morphine spontaneous withdrawal state on P14 could mimic USV spectrotemporal effects that are consistent with a developmental delay.”

Discussion section:

"Emission of USVs by pups is exhibited during distress (Hofer, 1996) and typically peak at P7 and then decrease significantly by P14 (Elwood and Keeling, 1982).”

Fig 4 is very unclear in that the y-axis has USV total distance, or distance/min. What exactly is being quantified here? The figure does not seem to fit with the legend.

RE: We apologize for the mismatch between the legends and figures. Fig. 4A is total distance over 10 min during USV recording which we now indicate in the y-axis. For Figure 4B and 4C, it is distance (m) per min over 10 min. Total distance was quantifed in meters (m) in AnyMaze.

I was very confused by the figure legend vs figures for Fig 7. The legend describes locomotor activity, but the figure portrays a number of Pearson’s’ correlation plots. There is also no reference to Fig. 7 in the text of the manuscript. With respect to the Pearson’s correlation s., I really don’t understand what information the correlations will provide comparing Saline P7 and morphine P14. Or saline P7 females and morphine P14 male??

RE: We again apologize for the mismatch between the legends and figures. We hope that the revised legends and figures are now clear and aligned correctly.

With respect to the Pearson’s correlations, we used unsupervised analysis to characterize the spectrotemporal patterns of USVs. This approach is informative because averaging the mean frequency of USVs, for example, does not capture the rich complexity of these signals. We use the Pearson’s correlations to show how similar or different the USV spectrotemporal patterns were between groups. Using the Pearson matrices, we used the mean values of the diagonals to make comparisons between groups and to perform statistical analysis. Previous studies showed that spectrotemporal USV patterns in mice are developmentally regulated. To permit assessment of these patterns during the P7-P14 developmental period, we chose saline P7 females as a reference group, which allowed us to compare this group to both P7 treatments (saline and morphine-exposed), and to the male and female P14 groups.

This approach allowed us to make developmental comparisons. For example, the Pearson correlations between the reference group and the saline P7 males was very high (Figure 6I, Left), indicating a high degree of similarity in the spectrotemporal USV patterns between these two groups. We also show that the Pearson correlation values between the reference group (P7 saline females) and morphine-exposed P7 groups is high, suggesting that prior morphine exposure does not seem to change the USV spectrotemporal patterns at P7 (Figure 7A and B, and 7G). We could have made one single saline P7 group combined from females and males (which would show qualitatively similar results), but, having a separate P7 saline reference group (females) allowed us to perform a developmental analysis of the USVs.

Figure 6 and 7 are included to illustrate the developmental regulation of USV profiles. Because the P7 saline females are so similar to the P7 saline males with regard to the spectrotemporal profiles (Pearson’s r = 0.9, Fig. 7G), we took advantage of this similarity and assigned the P7 saline females as the reference groups so that we could examine developmental regulation over time from P7 to P14 in saline control conditions. In the matrix, you can see that there are not that many blue values (low correlations) in the diagonal (small square, Fig. 6I). Next, when you compare to saline P14 groups, there is a dramatic decrease in Pearson correlation values (the values decrease across the diagonal, now you see a larger square in the matrix). This is shown in Figure 6I, Right Panel, and Figure 7C and Fig. 7G. This suggests that the USV spectrotemporal patterns have changed and are developmentally regulated.

The second question was how do the spectrotemporal USV patterns change with opioid exposure? When you compare our reference to the opioid P14 males and females, the data shows that the Pearson values drop, but this change is not significant (Fig 7G). Additionally, you can see in Figure 7D and E, that the values drop (blue values in matrices, squares) but not as dramatic as the saline P14 groups. The Pearson Correlation values for the opioid P14 groups (obtained from the diagonals) are also not different from either the P7 male group (relative to reference, high correlation values, (Fig 7G)) or the saline P14 groups (relative to reference, low correlation values, (Fig 7G)) This suggests that the USV spectrotemporal patterns in the P14 opioid-exposed group have similarities to both saline P7 and P14 groups. Because USV spectrotemporal patterns are developmentally regulated and in opioid-exposed mice the USV spectrotemporal patterns did not fully transition to the patterns observed at P14, one interpretation is that opioid exposure during early postnatal development leads to a developmental delay. The other interpretation is that the state of spontaneous opioid withdrawal mimics the less developed, P7 state.

The RNA seq analysis is a nice addition, however the fact that all genes with an unadjusted <0.10 were used for analysis is curious. P<0.05 is typically used. Can the authors justify?

RE: We initially used the more typical p<0.05 threshold for our enrichment analysis. However, given that the nature of this analysis was exploratory and given that our small sample size yielded a relatively small set of DE genes using the p<0.05 criteria (139 genes), by laxing the threshold to p<0.10, we included a larger gene set (370 genes) that enabled the detection of additional enrichment terms. We explicitly acknowledge that our sample size is underpowered; these results are intended to serve as a hypothesis-generating tool for future studies, not just at the individual gene level but at the level of biological pathways and gene networks.

The paper would have been greatly strengthened by a few validations of genes that were highlighted in the discussion. These genes could have major implications in our understanding of what is happening at a molecular level following neonatal opioid exposure and withdrawal, but the fact that nominal P values were used combined with lack of validation lessens the impact of this data.

RE: In the discussion of the revised manuscript, we report qPCR data for 6 genes that we chose based on their p-value, their level of expression, and our own biased interest based on the literature. These genes included Slc6a2 (NET), Prkcg, Hist1h2bc, Magel2, Onecut3, and Gabra6. We have added these data in Extended Data Figure 13-1. The general lack of correspondence between RNA-seq and qPCR is not surprising, for several reasons indicated in the Discussion section.

The bulk of this paper focuses on the establishment of the behavioral model; the addition of RNA-seq is just the first preliminary step in transcriptome analysis as we attempt to secure funding to conduct well-powered, comprehensive -omics studies across multiple tissues and cell types.

Reviewer 2:

Overall, this study is an examination of the effects of early-life opioid exposure in the mouse, as a model of neonatal opioid withdrawal syndrome (NOWS) on neurodevelopmental outcomes including USVs, pain reflexes, and later anxiety-related behavior, as well as an investigation into the transcriptomic consequences of this exposure. Few models exist to aid our study of the neurobiological impact of early-life opioid exposure. This study presents a new model, and provides neurobehavioral benchmarks of opioid exposure and their transcriptomic correlates.

Specific comments:

Methods:

1. Did the power calculations described in the methods include sex as a factor such that 6/sex/Tx is sufficient to observe any sex x drug Tx interactions?

RE: No, an n of 12 (6 per sex) was based on power analysis of the sex-averaged thermal nociceptive response on the hot plate on P7 (there were no main effects of Sex or interactions in the hot plate response). We have clarified this detail in the Methods section:

"A power analysis based on the effect size of the sex-averaged thermal nociceptive response on the hot plate on P7 in morphine-exposed mice versus saline control mice (Cohen’s d = 1.55) was used to estimate the required sample size using a two-tailed t-test in G*Power (Faul et al., 2007). A sample size of 12 per Treatment provides 95% power (p < 0.05). Thus, for the behavioral studies, we employed a minimum of n = 12 per Treatment (6 females, 6 males).”

2. Were the randomly assigned saline-tx pups within-litter controls? Or did all pups in a litter receive the same drug exposure? Clarity in the methods would be helpful to understand if litter effects or litter size need may be a concern.

RE: We used a mixed-treatment litter design whereby one-half of the pups were randomly assigned saline treatment and the other half was randomly assigned morphine treatment. We have further clarified this in the Methods section as follows:

"We employed a mixed-litter design whereby one-half of the pups were randomly assigned to saline (20 ml/kg, s.c.) and the other half randomly assigned to morphine (15 mg/kg, s.c.) within the same litter. The rationale for choosing a mixed-litter design was to avoid introducing confounds between litters that could skew the results (e.g., genetic relatedness, maternal behavior, or the general cage environment).”

3. Previous research has demonstrated that sex of the experimenter can influence behavioral performance of rodents in a sex-dependent manner (e.g. Sorge et al, Nature Methods, 2014; Bohlen et al, Behav Brain Res, 2014). Reporting the sex of the experimenter here would aid in reproducibility and comparisons with other models and work in this area.

RE: The experimenters who collected the behavioral data were all females. We have added the following to the Methods:

"All behavioral data were generated by female experimenters, thus controlling for the sex of the experimenter as a source of variance (Bohlen et al., 2014; Sorge et al., 2014).”

4. Was the temperature of the pups collected at the time of USV recording? Temperature is known to increase USV production - a cold pup will produce more calls to elicit retrieval and care from the dam (Branchi et al., 1998, PMID 9810475). It would be helpful to show here if the increase in call rate in the morphine-treated pups was secondary to possible temperature dysregulation.

RE: No, we did not measure the temperature of the pups, but we will in the future. We acknowledge that the increase in USVs overall may depend on a drop in body temperature across all groups and that the more pronounced increase in USVs in morphine-exposed mice may require this stressor in order for an effect of Treatment to be observed. We have noted this as a limitation in the Discussion as follows:

"Despite keeping the pups warm between behavioral testing using an electric heating pad, one limitation of this study is that we did not monitor the body temperature of the neonates throughout testing. Because hypothermia in neonatal body temperature is known to induce USVs from the pups (Branchi et al., 1998) and because opioid withdrawal in mice (Belknap, 1989) and neonatal rats (Thornton and Smith, 1998) has been shown to be is associated with hypothermia, it is possible that the detection of the effect of prior morphine exposure on USV phenotypes depends on the presence of withdrawal-induced hypothermia to evoke USV responses.”

5. Clarity is needed in the statistical section. The fit of the distributions with the assumptions of univariate analysis needs to be reported to support the use of the parametric testing in the absence of transformations. In addition, the term “mixed” ANOVA was used indicating random and fixed effects were included in the model. If a true mixed ANOVA was used, please indicate which factors were treated as random. Figure 1 legend suggests that Treatment was a random factor - and while this was randomly assigned, in the statistical model it is likely more appropriate to treat it as a fixed effect since those are the variables within a model that are expected to influence the outcome measure and random effects are those for which the variance attributed may need to be controlled (like batch effects or litter effects) but that aren’t expected to impact the outcome.

RE:

Thank you for these observations. In the revised manuscript, we assessed the residuals for normality using IBM SPSS Statistics 27. In the case of non-normal distributions (failed Shapiro-Wilk test for normality) we performed percentile rank normalization as described in Templeton 2011 (reference provided in manuscript). Following implementation of the normalization procedure, we then re-ran normality tests on the residuals to ensure the Shapiro-Wilk test was not violated (p>0.05) and evaluated the data for outliers (there were no statistical outliers in any of the datasets). Mauchly’s test of sphericity was performed for repeated measures with more than 2 levels (body weight and USV analysis). If the assumption of sphericity was violated (in all instances with Epsilon < 0.75), we report the Greenhouse-Geisser corrected statistics. Homogeneity of variances was confirmed using Levene’s Test of Equality of Error Variances.

We have thus edited both the statistical analysis and results sections to reflect these changes. The following was added to the statistical analysis section:

Statistical analysis

"Data analysis was performed in R (https://www.r-project.org/), IBM SPSS Statistics 27, and GraphPad Prism 8.0 (GraphPad Software). Body weight, hot plate, tail withdrawal, self-righting, USV/min, and USV distance data were analyzed using multifactorial RM ANOVAs, with drug Treatment and Sex as between-subjects factors and either Age/Day or Time as fixed effects repeated measures. Distance and percent center time in the open field were analyzed using 2-way ANOVA, with drug Treatment and Sex as independent variables. Body weight from P1 was also analyzed using a 2-way ANOVA due to partially missing data. Prior to all AVOVAs, data residuals for each phenotype were assessed for normality prior to parametric analysis. In the case of a non-normal distribution of the residuals (failed Shapiro-Wilk test for normality, p<0.05), data were percentile rank normalized as described (Templeton, 2011) and the Shapiro-Wilk test was repeated to confirm successful normalization (p>0.05). To facilitate interpretation of the data, normalized data are presented as raw measurement values. Mauchly’s test of sphericity was performed for repeated measures with more than 2 levels (body weight and USV analysis). If the assumption of sphericity was violated, Greenhouse-Geisser corrected statistics were reported. Homogeneity of variances was confirmed using Levene’s Test of Equality of Error Variances. Post-hoc comparisons were performed in the case of a significant interaction to determine group differences. Either t-tests (paired or unpaired) with Bonferroni correction or Tukey’s Honestly Significant Difference (HSD) tests were conducted for multiple comparisons.”

Modifications to the statistical results in the Results section and in the Figure Legends are included as blue text in the revised manuscript.

We removed the term “mixed-design” and have replaced with with simply, “RM ANOVA” in most cases. All repeated measures (RM) were treated as fixed effects (categorical); not random effects, as now indicated.

Treatment was never included as a random effect, nor were any other variables. When we used the term “random", we meant that sometimes, there can be unintended (a.k.a. “random", “spurious", “not biologically real", “by chance”) effects of treatment assignment on variables, in particular with small sample sizes. As an example, imagine if, by chance (low probability), we as the experimenters thought we were “randomly” assigning treatments prior to starting the experiments and it just so happened (by chance) that we assigned all of the heavy pups to one treatment group and all of the lightest pups to the other treatment group. This is all we meant by “random”.

We have changed the text in Figure 1 legend from:

"Thus, there was no random effect of treatment"

To:

"Thus, bodyweight was not significantly different between treatment groups prior to the first injections.”

Results:

1. Were there any deaths in the saline-treated group, suggesting deaths may be attributable to injection issues? If not, please justify the morphine dose used here. Is it translationally relevant? The pup deaths suggest it may be on the higher end? Are pup deaths typical at this dose at these ages?

RE: There were no deaths in the saline group.

We now state this at the beginning of the Results section:

"No deaths were observed in the saline group.”

Over the past 2-3 years, we have made adjustments to this model. Since this data was collected (roughly 2 years ago), we have switched to once daily injections of 15 mg/kg morphine sulfate pentahydrate and have largely replicated the phenotypes described in this manuscript while reducing the mortality rate associated with morphine exposure. There is no strong evidence supporting translationally relevant dosing, nor is there sufficient existing data to determine if the observed mortality rates are typical. Prenatal exposure in a clinical setting is highly variable, depending on the opioid(s) administered, dose, frequency, context (opioid maintenance versus illicit, intermittent high-dose misuse) and window of exposure during development. With that being sad, we consider our model as a reasonable starting point for determining the broad effects of opioid exposure, as well as opioid withdrawal, on development, behavior, gene expression, etc.

Note that the 15 mg/kg dose that we refer to is based on the total molecular weight of morphine + sulfate + pentahydrate and thus, the dose administered of morphine alone is closer to 5 mg/kg.

We now clarify this point in the Methods:

"The morphine dose is based on total molecular weight of morphine sulfate pentahydrate (not just morphine).”

2. No significant sex x treatment or sex x treatment x day interactions were reported in the Fig 1 legend. For clarity, it would be helpful to add a statement to the results indicating sex did not interact with treatment to influence weight gain.

RE: We now report the null result for the three-way interaction in the Figure 1B legend that justifies presenting the sex-combined data for body weight across days as follows:

”...but no Treatment x Sex x Day interaction (F1.36,97.97 = 0.07; p = 0.86); thus, the sex-combined data are presented.”

3. For the righting reflex data, is there justification for examining sex x treatment effects on P7 independently or within sex & treatment comparisons across ages since the three-way interaction of sex x treatment x day was not statistically significant in the omnibus model? If these were post hoc analyses, was a correction applied for multiple comparisons? In addition, the variance within the females (and the saline-treated groups on P4) seems large. Was there homogenous variance between groups? If not, might a non-parametric or transformation be appropriate?

RE: After re-running our analyses, we found the self-righting data was not normally distributed. After percentile rank normalizing the data (and confirming normality with a Shapiro-Wilk Test), we re-ran the RM ANOVA. We subsequently found significant Treatment x Sex and Treatment x Sex x Day interactions. We additionally confirmed homogeneity of variances post-normalization with Levene’s Test. We added the following to the Fig. 2 legend.

"In examining the normalized latency to self-right on P4 and P7, there was a main effect of Postnatal Day (F1,32 = 18.61, p < 0.001), Treatment (F1,32 = 49.23; p < 0.001), Sex (F1,32 = 8.30, p = 0.007), a Treatment x Sex interaction (F1,32 = 4.29, p = 0.046), and a Treatment x Sex x Day interaction (F1,32 = 4.69; p = 0.038).”

4. In lines 249-250, the sentence describing the lack of sex effects within each treatment group across time on USVs on P14 reads like there is no difference between groups with the wording “compared to the saline group”. Rewording this sentence would help clarify that the treatment effect was strong and sustained, but there was no interaction with sex at this age.

RE: We have re-worded the sentence as follows (see also the response to the next comment (5) regarding analysis of USV data):

"On P14, we detected significant elevations in USVs/min in morphine-treated pups during min 4 and mins 6-10 of the recording session (Fig.3C). Unlike on P7, we detected no significant effects of/interactions with sex on P14.”

5. For the USV data in Figure panel 3B, what is the justification for examining Treatment x Sex x Minute effects without a significant 3-way interaction?

RE: Please note that after re-running our analyses, we found the USV/min data was not normally distributed. After percentile rank normalizing the data (and confirming normality with a Shapiro-Wilk Test), we re-ran the analysis (RM-ANOVA). We subsequently found a significant Treatment x Sex x Time interaction which led us to re-run the RM-ANOVAs for each sex individually. We edited the legend of Figure 3B with the updated statistics as follows:

"(B): Greenhouse-Geisser corrections were applied when examining normalized USVs per min on both P7 and P14 (Mauchly’s Test; P7: χ244 = 117.39, p < 0.001, ε = 0.69; P14: χ244 = 155.41, p < 0.001, ε = 0.60). On P7, and there was a Treatment x Sex x Time interaction (F6.22,435.22 = 2.34; p < 0.03). Subsequent analysis of female-only data revealed a significant Time x Treatment interaction (F6.07,230.58 = 6.91; p < 0.001), driven by elevated USVs per minute during the first 3 min in morphine females relative to saline-control females (* Bonferroni adj. p = 0.004, 0.009, and 0.02, respectively). In contrast, male-only analysis did not yield a significant Time x Treatment interaction (F5.29,169.19 = 0.83; p = 0.54), only a main effect of Time (F5.29,169.19 = 3.54; p = 0.004).”

6. For USV syllable repertoire data in Figure 4, in the legend it is stated that there were significant effects of Development x Group Factors for 5/6 outcomes. For Frequency Bandwidth, what is the justification for examining within Development (which we assume is age here?) differences across sex & treatment since the interaction was not significant? What is “Group Factors” here. Does that mean a predictor with 4 levels - F Mor, M Mor, F Sal, and M Sal? Why not add sex and treatment as their own factors in this model? That might provide interesting interactions between sex and treatment that cannot be observed with a 4-level factor. The use of the term “sex-specific differences” in line 261 doesn’t seem appropriate without sex x treatment analysis.

RE: We have re-run this analysis and performed normalization as described above prior to parametric analysis with RM-ANOVA. We have included Postnatal Day, Treatment, and Sex as factors. Please refer to the updated results and Figure 5 legend. We have also updated Figure 5 to only include post-hoc comparisons for significant interactions (namely significant Treatment by Age interactions in panels A-E; comparisons performed to determine Treatment-driven differences on P7 and P14). There were no effects of Sex or interactions with Sex in our updated analysis, but we have elected to show the sexes individually to clearly illustrate this lack of sex-specific differences.

7. The correlation of syllable types between groups to show sex differences is a really interesting and informative way to approach these data.

RE: Thank you.

8. For the correlation data in Fig 6G, why was a one-way ANOVA used over a three-way ANOVA with sex, treatment and age as factors? If these are the same mice recorded at both P7 and P14, then the data are likely not independent and a within-subjects factor may be necessary.

RE: In Figure 5 of the revised manuscript, when we examined the effect of repeated morphine exposure on USV spectrotemporal patterns, we did not identify any main effects of Sex or interactions of Sex with other variables.

Therefore, In Figure 6 and 7 of the revised manuscript, and for purposes of statistical analysis, we grouped females and males into one cohort. In Figure 6, we asked whether USVs are developmentally regulated, to test this we performed a simple t-test to compare the Pearson’s correlation values (obtained from the diagonal of the matrices) obtained from comparisons between P7 saline males and P14 saline groups to the reference groups (P7 saline females). In Figure 7, we used a similar approach and performed a two-way ANOVA to examine the effects of Postnatal Day, Treatment, and Postnatal Day x Treatment interactions. We used this statistical approach to show that the effects of repeated morphine exposure depend on Postnatal Day, with P14 groups being more affected by repeated morphine exposure relative to morphine P7 groups.

9. The ANOVA statistics should be included in the Figure legends 6G and 5J.

RE: We added the statistics in the revised version of the manuscript (Figures 5, 6, and 7); please see the figure legends.

10. For the open field data, why was an Unpaired t-test conducted between the treatment groups for percent time in center when the Treatment effect in the ANOVA was significant and already revealed this effect?

RE: We removed the t-test and left the main effect as a near-significant result. In the new analysis of normalized data, this effect did not reach statistical significance (p=0.053).

11. The interpretation of the open field decreased percent time in center by the MOR animals as increased anxiety-related behavior is confounded by the overall hypoactivity. Because the mice traveled less around the arena, as a result they are in the center area less - even showing these data as percent of total time in the center. I think the interpretation here needs to be coupled with acknowledgement of the confound.

RE: We now explicitly note the confound of locomotor activity with the measurements that are considered anxiety-like.

We changed the title of the figure to simply read:

"Decreased locomotor activity in the open field at P21 following repeated neonatal morphine exposure.”

We added this text to the Results:

"Both of these effects could reflect increased anxiety-like behavior and/or decreased exploratory behavior resulting from early postnatal administration of morphine.”

We changed this text in the Discussion:

"We found several important NOWS-related phenotypes in female and male mice, including reduced weight gain, developmental motor delays and USVs, agitation and decreased locomotor activity in the open field test of anxiety-like behavior, and thermal hyperalgesia.”

We changed Figure 1A:

(A): Mice underwent assessment for USVs and thermal nociception. A subset of mice also underwent self-righting assessment at P4 and P7 and testing for locomotor activity and center time in the open field on P21.

We changed Figure 9B

"We note that the decreased locomotor activity in the morphine-exposed mice could concomitantly affect overall time spent in the center and thus, by extension, could affect % open field center time as an anxiety-like measure.”

Minor comments:

1. The word “Opioid” is missing from the NOWS definition in line 31.

RE: Fixed, thank you

2. The word “during” is missing from line 99 before “behavioral testing”.

RE: Fixed, thank you

3. Line 228 uses the word “lead” instead of “led”.

RE: Fixed, thank you

4. Increasing the font size of the syllable plots in Figure 5 would increase legibility.

RE: Since we had to fit so many syllable repertoires into the figure, it is difficult to accommodate a larger font size. As legibility is not vital to the interpretation of our results, we kept the font size the same.

5. Line 266 - is this Fig 6C of 5C?

RE: Fixed, thank you

6. The figure number citations appear to be mixed up between the number they are given in the manuscript, cited in the text and labeled in the legends.

RE: We apologize and have addressed all inconsistencies between figure call-outs and figure numbers.

7. The panels for Fig 8 are not described in the correct order - B is not described and C is labeled as B.

RE: We have changed the order of description to match the order of data presentation for Figure 8.

8. A word is missing from line 375 “then by P14”.

RE: and then decrease significantly by P14

9. Line 369 is missing “by”.

RE: We have changed this to:

"Overall, female mice were more vulnerable to the effects of morphine during P1-P14 based on these neurobehavioral and development phenotypes.”

We thank the reviewers for careful critique of the manuscript.
